# Single-Cell Transcriptomic Landscape of the Gill in Hypoxia-Stressed *Tachysurus vachellii* (Richardson, 1846): Insights into Cell-Type-Specific Responses of Different Epithelial Cells and Erythrocytes

**DOI:** 10.3390/cells15191747

**Published:** 2026-09-24

**Authors:** Xiaoyun Wu, Yeyu Chen, Feiyang Li, Luyun Ni, Pengcheng Li, Quan Gong

**Affiliations:** 1 Fisheries Research Institute, Sichuan Academy of Agricultural Sciences/Sichuan Fisheries Research Institute, Chengdu 611731, China; 2 Fish Resources and Environment in the Upper Reaches of the Yangtze River Observation and Research Station of Sichuan Province, Chengdu 611731, China; 3 Fishes Conservation and Utilization in the Upper Reaches of the Yangtze River Key Laboratory of Sichuan Province, Chengdu 611731, China

**Keywords:** scRNA-Seq, neuroepithelial cells, cell communication, transmission electron microscope

## Abstract

The study aims to construct a single-cell atlas of the gill of yellow catfish *Tachysurus vachellii* (Richardson, 1846) and to explore the response patterns of gill cells under acute hypoxia stress. In this study, we performed single-cell sequencing and histological observations of *T. vachellii,* gills. A total of 37,090 cells and 33 cell clusters were collected, and 18 cell types were identified. We found that three clusters of NECs have different functions, and in the gene enrichment results of MC differential expression during hypoxia and reoxygenation, we observed that MC may be regulated by non-neuronal neurotransmitter signals during hypoxia stress. There are also differences in cellular communication and transcription factors between different NECs and MCs. After acute hypoxia, the expression of *tjp2* and *lima1* in PVC was significantly upregulated, and there was no significant change in the number of erythrocytes. Observation of the organizational structure revealed that muscle, skin, and gill tissues were affected after hypoxia, resulting in changes in the flattened epithelial cells and MRC ultrastructure of the gill. This study provides single-cell atlas and data support for the acute hypoxia and reoxygenation model of fish gill tissue.

## 1. Introduction

Hypoxia has always been one of the most important environmental factors that restrict the survival and development of aquaculture industry [1]. Apart from the influence of climate and weather, intensive aquaculture itself, with its high density, high feeding, and high yield, makes farmed fish extremely vulnerable to hypoxia challenges. The most obvious phenomena after hypoxia are poor appetite, slow growth, and weight loss [2,3]. Long-term hypoxia leads to a decrease in immune factors and immune cell activity, which suppresses immune function, reduces resistance, increases the risk of disease, and, in severe cases, can lead to death [4]. Therefore, the study of hypoxia in fish has always been a research area of high interest in the aquaculture industry.

There is a wealth of academic research on growth, enzyme activity, metabolism, transcriptome, and other aspects. Single-cell RNA sequencing (scRNA seq) has gradually emerged as one of the most advanced technologies in recent years; it is capable of analyzing transcriptome molecular features at the single-cell resolution level. The core breakthrough lies in overcoming the limitations of traditional bulk sequencing, which mixes millions of cells and takes the average, thus revealing the masked heterogeneity in cell populations. In the study of aquatic animals, scholars have applied single-cell transcriptome sequencing technology to draw cell maps, such as the skin cell map of the long snouted catfish [5], the spleen immune cell map of zebrafish [6], and the kidney cell map of Atlantic salmon [7].

Yellow catfish is one of the most economically important fish species in China; it is well-known for its high nutritional value and soft and tender taste. In addition, China has a huge aquaculture industry for yellow catfish, so it is crucial to conduct research on hypoxia stress in yellow catfish and decipher the molecular and cellular mechanisms of hypoxia tolerance for selective breeding and aquaculture management. A large number of researchers have conducted studies on the hypoxic stress of yellow catfish, involving the effects of hypoxia on the digestive enzyme activity of *Tachysurus fulvidraco* (Richardson, 1846) [8], liver metabolism [9], and immune antioxidant effects of *T. vachellii* [10]. As the main organ for gas exchange, ion regulation, and immune monitoring, gills are the first line of defense against environmental fluctuations [11]. After hypoxia exposure, fish gills undergo significant morphological and functional remodeling, including lamellar changes [12], chloride cell proliferation [13], and vascular regulation [14]. In particular, the neuroepithelial cells (NECs) distributed along the gill filaments serve as specialized oxygen sensors and can regulate ventilation function under hypoxic conditions [15]. At present, there is a lack of research on the differences in the molecular expression of various cell-related signaling pathways in the gill tissue of yellow catfish after acute hypoxia stress. In this study, we established an acute hypoxic stress model for the yellow catfish and used single-cell transcriptome detection technology to construct its gill cell atlas. At the same time, we explored the relevant response mechanisms of cells such as neural epithelial cells, red blood cells, and mucous epithelial cells, laying a data foundation for single-cell hypoxia research.

## 2. Materials and Methods

### 2.1. Experiment and Sample Collection

The experimental fish were provided by the Fisheries Research Institute of the Sichuan Academy of Agricultural Sciences. After two weeks of acclimation, healthy one-year-old *T. vachellii* with a body weight of 51.04 ± 3.31 g and a body length of 15.66 ± 0.58 cm were selected for a preliminary hypoxia experiment to determine the hypoxia tolerance parameters. At a dissolved oxygen (DO) level of 2.00 mg/L, the fish began to gasp at the surface; when DO continuously dropped to 0.55 mg/L, signs of asphyxiation appeared. Consequently, the experimental DO was set at 0.75 mg/L to ensure that the fish were in a hypoxic condition and could also survive throughout the trial period. We established three experimental groups: control (C: DO 6.5 ± 0.1 mg/L), hypoxia (H: DO 0.75 ± 0.10 mg/L for 6 h), and recovery (R: DO 7.0 ± 0.2 mg/L for 6 h and 24 h). We prepared three glass tanks (100 cm × 50 cm × 35 cm fiber glass tanks), with 12 male fish randomly placed in each tank. We randomly selected one fish from each tank at 0 h for sampling (C), and then took samples in the same way at 6 h of hypoxia (H), 6 h of oxygen restoration (R), and 24 h of oxygen restoration (R24).

After the fish were anesthetized with MS222 (60 mg/L, buffered with sodium bicarbonate, pH 7.0–7.5.), gills from three fish per group were taken at C, H, and R for single-cell RNA-seq; gills, skin, and muscle were sampled from C, H, R, and R24 fish for histological observation; gills from the same four time points were processed for ultrastructural observation. (For the R24 group, only histological observations were conducted. Our experimental design focused on acute hypoxia and short-term recovery. Based on the preliminary results, the physiological state of the fish had largely recovered by 24 h of reoxygenation; therefore, single-cell sequencing was not performed at this time point.)

### 2.2. Preparation of Single-Cell Suspension

The excised gill tissue was rinsed with Hanks solution (Gibco, Grand Island, NY, USA), minced, digested with collagenase (Gibco, Grand Island, NY, USA), and passed through a 70 μm cell strainer to remove undigested fragments; the filtrate was centrifuged at 350 *g* for 5 min at 4 °C, the supernatant discarded, and the resulting pellet resuspended to obtain a single-cell suspension.

### 2.3. Library Construction and Single-Cell RNA Sequencing

A small amount of single-cell suspension was added to an equal volume of 0.4% trypan blue staining solution, and the Countess^®^ II Automated Cell Counter (Invitrogen, Thermo Fisher Scientific, Waltham, MA, USA) was used to adjust the concentration of live cells to the ideal concentration (1000–2000 cells/μL) for cell counting. The gel beads containing barcode information combined with the mixture of cells and enzymes, entered the reservoir, and were separated by oil to form GEMs (Gel Beads In Emulsions). After that, the gel beads dissolved and released the captured sequence containing the barcode information; the cDNA fragment was reverse-transcribed, and the sample was labeled. The gel beads and the oil drops were broken, and the cDNA was used as the template for PCR amplification. All GEM products were mixed, and the standard sequencing library was constructed. Firstly, the cDNA enzyme was cleaved into fragments of about 200–300 bp, followed by conventional second-generation sequencing library construction steps such as end repair, A-tail addition, sequencing adapters P5, P7, and sample index. Finally, PCR amplification was performed to obtain the DNA library. High-throughput sequencing was performed on the constructed library using the PE150 sequencing mode of the Illumina sequencing platform (10× chemistry/version: Chromium NextGEM Single Cell 3′HT Kit v3.1 (10× Genomics, Pleasanton, CA, USA)).

High-throughput sequencing was performed on the final libraries using Illumina NovaSeq X Plus (Illumina, San Diego, CA, USA). A Single Cell 3′ Library comprised standard Illumina paired-end constructs which begin and end with P5 and P7. The Single Cell 3′ 16 bp 10× Barcode and 10 bp unique molecular identifier (UMI) were encoded in Read 1, while Read 2 was used to sequence the cDNA fragment. Sample index sequences were incorporated as the i7 index read. Read 1 and Read 2 were standard Illumina^®^ sequencing primer sites used in paired-end sequencing [16].

### 2.4. Sequencing Data Analysis

We used the official 10× Genomics analysis software Cell Ranger 8.0.0 to perform data quality statistics on the raw data and compared it to the reference data (unpublished). Using Cell Ranger, we proceeded to filter and correct barcodes and UMIs. Then, by deduplicating the UMIs associated with each gene ID for every barcode, the number of unique UMIs were counted as the expression level of that gene in the corresponding cell. To further capture valid cells with low RNA content, Cell Ranger employed an EmptyDrops-based approach to identify genuine barcodes. The number of genes identified in single cells was 200.0–3200.0. The total number of UMIs in single cells (less than 16,000.0) should be excluded; doublet-removal method: doubletfinder; normalization: LogNormalize; batch-correction procedures: harmony; number of PCs: 50; clustering resolution: 0.5.

After gene expression quantification with Cell Ranger, we transferred the expression matrix to Seurat 5.1.0 [17] for downstream analysis. Seurat uses a graph-based clustering algorithm to partition all cells in the scRNA-seq dataset into distinct subpopulations, facilitating downstream analyses. It then applies the non-linear dimensionality-reduction technique t-SNE (t-distributed Stochastic Neighbor Embedding) to project the high-dimensional transcriptional profiles into a two-dimensional space, placing cells with similar expression patterns close together. Finally, cell types were assigned by comparing the expression profiles of query clusters against reference signatures.

To compare cell subpopulations between groups, a pseudo-bulk approach was employed, and differentially expressed genes (DEGs) were identified using edgeR 3.42.4. Genes were considered differentially expressed when they met the following criteria: (1) log2-fold change ≥ 1 (i.e., ≥2-fold upregulation) and (2) *p*-value ≤ 0.05. The differentially expressed genes obtained were further analyzed by Gene Ontology (GO) and Kyoto Encyclopedia of Genes and Genomes (KEGG) enrichment analyses. Gene Set Enrichment Analysis (GSEA) was performed to assess whether predefined functional gene sets were globally upregulated or downregulated between specific groups.

All transcription factors expressed in the dataset were annotated against the animalTFDB database. We used the scenic R software 1.3.1package to infer the transcription factor network, and the AUCell R software 1.22.0 package to score the activity of each regulator in a single cell based on AUC values [18].

We analyzed the communication ligand–receptor pairs and their expression information between cell subpopulations using CellChat V2.1.0 software, and constructed a cell interaction network diagram at the ligand–receptor level to predict potential communication network relationships between small cells [19]. The mapping was completed by homologous alignment using zebrafish ligand–receptor gene pairs. The zebrafish ligand–receptor gene pairs were derived from single-cell transcriptomics analyses of neural stem cell heterogeneity and contextual plasticity in a zebrafish brain model of amyloid toxicity; the corresponding ligand–receptor gene pair website is ligand–receptor connectome.

### 2.5. Histological Observation

Tissues were fixed in 4% paraformaldehyde (Servicebio, Wuhan, China), dehydrated (dehydration duration: 75% alcohol (Servicebio, Wuhan, China) 3 h, 85% alcohol 2 h, 95% alcohol 1 h, 100% alcohol I 20 min, 100% alcohol II, 100% alcohol III 20 min, 100% alcohol IV 20 min, transparent agent I 25 min, transparent agent II 30 min, paraffin I 30 min, paraffin II 1 h, and paraffin III 2 h), paraffin-embedded and sectioned (3 μm). Sections were dried at 60 °C for ≥2 h, stained with hematoxylin (Servicebio, Wuhan, China) for 10–20 min, rinsed in water for 1–3 min, differentiated in hydrochloric-acid (Servicebio, Wuhan, China) alcohol for 5–10 s, rinsed again for 1–3 min, blued in 50 °C warm water until a clear blue color appeared, and washed for 1–3 min. After 85% ethanol (3–5 min), sections were counter-stained with eosin (Servicebio, Wuhan, China) for 3–5 min, briefly rinsed (3–5 s), dehydrated, cleared, and mounted with neutral balsam. Slides were scanned using a digital slide scanner (3DHISTECH, Pannoramic 250, Budapest, Hungary).

### 2.6. Transmission Electron Microscopy (TEM) Analysis

Gills for TEM were pre-fixed in 3% glutaraldehyde (Servicebio, Wuhan, China) and subsequently (Sigma-Aldrich, St. Louis, MO, USA) post-fixation in 1% osmium tetroxide. After dehydration in an acetone series (acetone was dehydrated step by step, with a concentration gradient of 30% → 50% → 70% → 80% → 90% → 95% → 100%), and after preparing the dehydrating agent and Epon-812 (Polysciences, Warrington, PA, USA) embedding agent in ratios of 3:1, 1:1, and 1:3, respectively, the tissue was sequentially infiltrated, followed by pure Epon-812. Ultrathin sections (60–90 nm) were cut, collected on copper grids, and stained with 2% uranyl (Polysciences, Warrington, PA, USA) acetate for 10–15 min and then with lead citrate (Polysciences, Warrington, PA, USA) for 1–2 min. Cellular ultrastructure was examined under a transmission electron microscope (JEOL, JEM-1400FLASH, Akishima, Japan).

## 3. Results

### 3.1. Cellular Composition of the Gill of T. vachellii Under Hypoxia

Following single-cell RNA sequencing, an average of 398,717,020 reads per library were obtained (Table S1, Genome Sequence Archive (GSA): CRA049483). In total, 37,090 cells were recovered across the three groups: C (3386, 3799, and 2286), H (2653, 6435, and 4898), and R (6977, 3791, and 2865). After Seurat-based quality control and filtering, 33,417 high-quality cells were retained, with a median gene count of approximately 874 per cell and a UMI count of approximately 1817. These cells formed 33 transcriptionally distinct clusters (cluster 0–32) following batch-effect correction and graph-based clustering (Figure 1A). By comparing cluster-specific marker genes, we identified the following cell types among the 33 clusters: ionocytes, mucous cells (MCs), pavement cells (PVCs), NECs, tuft cells (TCs), B cells, plasma cells, T cells, macrophages, myeloid cells, naive T cells, NK cells, dendritic cells, endothelial cells, erythrocytes, immune cells, platelets, proliferating cells, fibroblasts, epithelial cells, and a minor fraction of unassigned cells. The frequencies of cells across different groups are depicted in Figure 1B.

### 3.2. Single-Cell Transcriptomic Atlas of Gill Tissue in T. vachellii

We identified at least 18 distinct cell types in the gill tissue of *T. vachellii*, and the characteristic marker genes corresponding to each cell subset are detailed in Figure 1C and Supplementary Table S1. Among the 33 clusters, two that expressed *atp6v1f*, *atp6v1e1*, and *atp6v1a* highly were identified as ionocyte 1 (or H+-ATPase-rich (HR) cells) [20]. Another cluster, marked by high *atp1a1* expression, was identified as ionocyte 2 (or Na+/K+-ATPase-rich (NaR) cells) [21]. *Muc2*, *agr2*, and *fcgbp* were found to be highly expressed in three clusters, which were consequently identified as MCs [16,22,23]. Muc19, like muc2 and muc5, belongs to the large secreted gel-forming mucins [24]. Since *muc19* and *agr2* were co-expressed at high levels in cluster 14, we classified this cluster as MCs. Clusters 5, 6, and 16 were identified as PVCs, as they showed high expression of *cldn4* and *cldn8* [20]. Notably, *upk1a* was exclusively enriched in cluster 6 [25]. We identified two clusters of NECs based on the expression of *ddh*, *tph1*, and *sv2a* [21]. Cluster 30 simultaneously displays epithelial features (*cldn4* and *upk1a*), *sv2a* and the expression of *brn3*, a neuronal-related gene [21,26]; however, aside from *sv2a*, no other definitive marker genes were identified for this cluster, and it could therefore only be tentatively identified as NECs. Cluster 17 was identified as endothelial cells, owing to the high enrichment of *lyve5*, *eng*, *pecam1*, and *cdh5* (http://bio-bigdata.hrbmu.edu.cn/CellMarker/, (accessed on 16 January 2026)). Fortunately, we also identified a cluster of TCs based on the marker genes *pou2f3* [21], *avil* [21], and *ehf* (http://bio-bigdata.hrbmu.edu.cn/CellMarker/). Cluster 24 was identified as erythrocytes based on the expression of the marker genes *hbb* [27], *alas2* [27], *ank1* [27], and *tfrc* [5]. Platelets were identified by the marker genes *itga2b*, *gp1ba*, and *itgb3* [28]. *Clec3a* [29], *col1a1*, *col1a2*, *col12a1*, and *dcn* [20] are regarded as fibroblast marker genes. Two further epithelial populations, Cluster 10 and cluster 12, were distinguished by selective enrichment of *krt13* and *krt17*, respectively.

B cells were identified by the marker genes *cd79a*, *cd79b*, and *pax5* [20]. Cluster 7 was further characterized as plasma cells owing to its abundant expression of immunoglobulin genes (*igkc2*, *iglc3*, *ighv4–59*, and *iglv3–19*) [25]. Macrophages were identified by the expression of the marker genes *csf1r* [30], *c1qb* [31], *c1qa* [7], and *c1qc* [31]. *Cd3e* [20], *lck* [20], *tox* [5], and *cd3d* [20] are canonical T cell marker genes. Dendritic cells were identified on the basis of *shtn1*, *znf366*, *tiam1* [5], and *cd74* expression (http://bio-bigdata.hrbmu.edu.cn/CellMarker/). *Nfkβia* and *faslg* are marker genes of NK cells [32]. Cluster 26 was identified as naïve T cells based on the expression of *tcf7* and *cd40lg* [32]. Cluster 1 was classified as myeloid cells due to its expression of marker genes (*ilr2*, *epx*, and *fabp4*) characteristic of multiple immune cells. In addition, we identified two further immune clusters (putative): one expressing *fcer2* and *lilra4*, and the other expressing *spic*. Two clusters, Cluster 3 and Cluster 29, remain undefined.

### 3.3. Effects of Hypoxia on Gill NECs in T. vachellii

We identified three NEC clusters: compared to the control group, the H group exhibited 141, 386, and 188 DEGs in NEC1, NEC2, and NEC3, respectively (Figure 2A and Tables S2–S4); the R group exhibited 104, 464, and 157 DEGs versus control; R vs. H comparisons revealed 150, 382, and 120 DEGs in the three NEC subtypes. The Venn diagram of DEGs in the NEC1, NEC2 and NEC3 groups is shown in Figure 2B. Taking the DEGs between H group and C group as an example, there were two genes simultaneously expressed in the three clusters of genes; 127, 357 and 169 genes were expressed separately. The DEGs among the groups were all expressed in NECs, as shown in Figure 2C. The Venn diagrams of the DEGs in the other groups are shown in Figure S1A,B, and partial DEGs of NECs are shown in Figure S1C.

Additionally, we analyzed transcription factors in three clusters of NECs. A total of 65 regulons were identified. These regulons contained 1237 target genes and 45 transcription factors. Bubble charts of the top three differentially enriched regulons identified among the three NECs are shown in Figure 3A, and UMAP projections depicting the mean regulon activity, binary activity and gene expression of *brn3* extended (15 g), *tead1* extended (27 g), and *nfatc3* extended (20 g), the top three differentially enriched regulons identified within each cluster, are provided in Figure S2. The heatmaps of mean regulon activity and binary activity in NECs after hypoxia and reoxygenation are shown in Figure 3B and Figure 3C, respectively. Notably, transcription factor and binary activity varied among the three clusters of NECs, suggesting distinct regulatory landscapes across clusters. The transcription factors with higher frequency of activity than group C in the three types of NECs after hypoxia were *bhlhe40* extended (12 g), *klf4* extended (29 g), *irf1* extended (22 g), *nfe2l2* extended (14 g) and *nfatc3* extended (20 g). Meanwhile, *irf1* extended (22 g) exhibited high frequency of activity in all three types of NECs.

### 3.4. Effects of Hypoxia on Gill Erythrocytes in T. vachellii

Compared to the C group, the H group exhibited 10 DEGs (five upregulated and five downregulated); the R group showed four DEGs (one upregulated and three downregulated); and the R versus H group comparison revealed five DEGs (three upregulated and two downregulated) (Figure 4A,E and Table S5). The GO enrichment plot of DEGs in erythrocytes is shown in Figure S3A. Compared to the C group, the top three enriched GO terms for DEGs in the H group were macrophage migration inhibitory factor binding, fructose–bisphosphate aldolase activity and aldehyde–lyase activity. Compared to the C group, the top three enriched GO terms in the R group were fructose–bisphosphate aldolase activity, aldehyde–lyase activity and MHC protein complex. Compared to the H group, the top three enriched GO terms in the R group were MHC class I protein complex, respiratory system process and smooth muscle tissue development. The KEGG enrichment bar chart of DEGs is shown in Figure S3B. Compared to the C group, the top three enriched pathways in the H group were antigen processing and presentation, pentose phosphate pathway, and fructose and mannose metabolism; compared to the C group, the top three enriched pathways in the R group were pentose phosphate pathway, fructose and mannose metabolism and glycolysis/gluconeogenesis; compared to the H group, the top three enriched pathways in the R group were antigen processing and presentation, Epstein–Barr virus infection and human T cell leukemia virus 1 infection.

### 3.5. Effects of Hypoxia on Gill MCs in T. vachellii

MCs displayed the most extensive transcriptional response among gill cell types: 563 DEGs (176 upregulated and 387 downregulated) were identified in H versus C; 640 DEGs (126 upregulated and 514 downregulated) in R versus C; and 341 DEGs (115 upregulated and 226 downregulated) in R versus H (Figure 4B,E and Table S6). GO enrichment analysis (Figure S4A) revealed distinct functional signatures: under hypoxia (HvsC), the top three enriched terms were inhibitory synapse, nitric oxide biosynthetic process and nitric oxide metabolic process, whereas in R versus C, they were the regulation of epithelial-to-mesenchymal transition, epithelial-to-mesenchymal transition and ribonucleotide binding, and in R versus H, they were cofactor metabolic process, corticosteroid hormone secretion and glucocorticoid secretion. The KEGG enrichment bubble plot of DEGs is shown in Figure S4B. Compared to the C group, the top three enriched pathways in the H group were estrogen signaling pathway, apelin signaling pathway and ErbB signaling pathway; compared to the C group, the top three enriched pathways in the R group were influenza A, NOD-like receptor signaling pathway and coronavirus disease—COVID-19; compared to the H group, the top three enriched pathways in the R group were circadian rhythm, glycolysis/gluconeogenesis and metabolic pathways. Finally, the GSEA of H versus C DEGs in MCs identified extracellular ligand-gated ion channel activity, potassium channel complex and voltage-gated potassium channel complex as the top three enriched biological processes (based on normalized enrichment score, NES) (Figure S4C).

### 3.6. Effects of Hypoxia on Gill PVCs in T. vachellii

Relative to the C group, the H group exhibited 771 DEGs; the R group showed 554 DEGs; and the R versus H comparison revealed 507 DEGs (Figure 4C,E and Table S7). Compared to the C group, the top three enriched GO terms for DEGs in the H group were vitamin binding, reactive nitrogen species metabolic process and the regulation of T cell apoptotic process; in the R group, they were immune system process, regulation of immune system process and immune response; in the R versus H comparison, they were lysozyme activity, muscle hypertrophy and striated muscle hypertrophy (Figure S5A). The KEGG enrichment bubble plot of DEGs is shown in Figure S5B. Compared to the C group, the top three enriched pathway in the H group were cell adhesion molecule (CAM) interaction, graft-versus-host disease and cytosolic DNA-sensing pathway; compared to the C group, the top three enriched pathways in the R group were leishmaniasis, C-type lectin receptor signaling pathway and the Toll-like receptor signaling pathway; compared to the H group, the top three enriched pathways in the R group were Toll and lmd signaling pathway, cytosolic DNA-sensing pathway, and steroid hormone biosynthesis.

### 3.7. Effects of Hypoxia on Gill TCs in T. vachellii

Compared to the C group, the H group exhibited 146 DEGs (64 upregulated and 82 downregulated); the recovery group showed 108 DEGs (46 upregulated and 62 downregulated); and the R versus H comparison revealed 163 DEGs (64 upregulated and 99 downregulated) (Figure 4D and Table S8). Compared to the C group, the top three enriched GO terms for DEGs in the H group were mitochondrial respiratory chain complex IV, nucleoside metabolic process and respiratory chain complex IV (Figure 4E); in the R group, they were catalytic activity, phosphotyrosine binding and the heparan sulfate proteoglycan biosynthetic process (Figure S6A); and in the R versus H comparison, they were ATP metabolic process, mitochondrial respiratory chain complex IV and the regulation of chromatin organization (Figure S6A). The KEGG enrichment bubble plot of DEGs is shown in Figure 4F and Figure S6B. Compared to the C group, the top three enriched pathways in the H group were spliceosome, TNF signaling pathway and legionellosis; compared to the C group, the top three enriched pathways in the R group were spliceosome, measles and oxidative phosphorylation; compared to the H group, the top three enriched pathways in the R group were glycolysis/gluconeogenesis, TNF signaling pathway and the biosynthesis of amino acids.

### 3.8. Cell Communication Analysis of NECs and MCs in Gill of T. vachellii

Due to the discovery of GO enrichment classification related to neural-related functions in the GSEA of MCs, we conducted cell communication analysis between NECs and MCs (Figure 5). Under normal circumstances (C group), the ligand–receptor pairs with significant communication probability and rankings in the top 30 are shown in Figure 5A. These interactions were predominantly associated with biological processes including cell adhesion (e.g., neuronal cell adhesion), tight junction formation, immune regulation, developmental signaling, and inflammatory responses. The top 15 signaling pathways with *p*-values among various cell subpopulations are shown in Figure 5B. From the five most significantly enriched pathways, we selected two neural-related pathways—ncam and notch signaling—for detailed visualization (Figure 5C). In the NCAM pathway, NEC1 and NEC2 exhibited communication with all three NEC subtypes (NEC1, NEC2, and NEC3). In the NOTCH pathway, NEC3 communicated with MC1, MC3, and MC4; MC4 reciprocally communicated with NEC1, whereas NEC2 only communicated with NEC1. 

The differences in the interaction between NECs and MCs between the H group and the C group are shown in Figure 6A. After hypoxia, the number of ligand–receptor pairs corresponding to MC2 and MC4 in MCs increased, while the number of ligand–receptor pairs for communication between NECs decreased. Through the corresponding heatmap (Figure 6B), we can more intuitively observe that the ligand–receptor pairs had the highest upregulation between MC4 and NEC1, and the highest downregulation between NEC2 and NEC3. The probability differences in ligand–receptor pairs in the ncam and notch signaling pathways after hypoxia are shown in Figure 6C,D. There were three cell pairs in group H with an upregulated ncam signaling pathway (ncam1b-l1cama), including NEC1 → NEC1, NEC2 → MC1, and NEC2 → MC4. There were 11 cell pairs in the H group notch signaling pathway that exhibited differences in communication probability and were all downregulated.

### 3.9. Effects of Hypoxia on the Tissue Histological Structure of T. vachellii

Histological sections of muscle, skin, and gills were examined (Figure 7). In muscle, the H group exhibited necrotic fibers with dissolved sarcoplasm and pyknotic and condensed nuclei, accompanied by marked lymphocyte infiltration (II stage lesion). In the skin, the epidermis was thickened in the H, R, and R24 groups (I stage lesion); swollen MCs were seen, and numerous melanocytes were present at the interface between the epidermis and the loose dermal layer. In the gills of the H group, we observed necrosis of epithelial cells, characterized by pyknosis, fragmentation, and cytoplasmic dissolution (I stage lesion).

### 3.10. Ultrastructural Alterations in Gill of T. vachellii Under Acute Hypoxia and During Reoxygenation

The TEM results of the gills of *T. vachellii* are shown in Figure 8. Under hypoxia, both PVCs and mitochondria-rich cells (MRCs) in the gills displayed ultrastructural abnormalities (Figure 8B,F). The mitochondria of PVCs were rod-shaped or circular, with a large number of broken and dissolved cristae, significant loss of matrix, local vacuolization, and continuous and intact outer membrane; damaged mitochondria accounted for an average of 67.68% (C group 2.08%), and the average nucleocytoplasmic (N/C) ratio was 18.16% (C group 57.26%). MRCs appeared to be undergoing apoptosis (nucleus not captured, so apoptosis could not be confirmed). In these cells, some mitochondria were swollen and ovoid; their cristae were extensively fractured and dissolved, the matrix was partially lost, electron density was reduced, and the outer membranes were ruptured; the average mitochondrial damage rate was 63.49% (C group 0%), and the average N/C ratio was 33.25% (C group 34.37%). After 6 h of reoxygenation, both PVCs and MRCs in the gill tissue showed only mild ultrastructural alterations. The mitochondria of PVCs showed mild swelling, with rod-shaped or circular morphology, partially broken and dissolved cristae, slightly reduced matrix, and slightly decreased electron density, while the outer membrane remained intact; the average mitochondrial damage rate was 11.11%, and the average N/C ratio was 51.68%. MRC nuclei were irregular or elliptical, with evenly distributed chromatin and intact nuclear envelopes; the perinuclear space was slightly widened; the average mitochondrial damage rate was 0%; and the average N/C ratio was 34.16%. After 24 h of reoxygenation, PVCs in the gill tissue showed normal morphology (the average mitochondrial damage rate and N/C ratio were 6.67% and 51.68%, respectively), while MRCs exhibited only mild abnormalities. MRCs lost cytoplasmic ribosomes and saw a decrease in cytoplasmic electron density (the average mitochondrial damage rate and N/C ratio were 0% and 45.14%, respectively).

## 4. Discussion

In fish, NECs embedded at the base of the gill filaments and lamellae have been shown to closely resemble the chemosensitive type I cells of the mammalian carotid body. Gill NECs can detect changes in O_2_ and CO_2_ levels in both the blood and the surrounding environment [33], sensing acute hypoxia within seconds and releasing neurotransmitters such as 5-hydroxytryptamine (5-HT) and acetylcholine (ACh) to trigger hyperventilation and increased cardiac output [34]. 5-HT, also known as serotonin, is a neurotransmitter released by presynaptic neurons and can be recycled via the serotonin transporter (Slc6a4) [35]. We identified three clusters of NECs in the gill tissue of T. vachellii, and our analyses suggest that these NECs may perform distinct functions. In NEC1, neural cell adhesion molecule 1 (ncam1) plays a crucial role in regulating neurogenesis and neuronal plasticity [36]. Two subtypes were identified on zebrafish, *ncam1a* and *ncam1b* [37], and performed different functions within the developing nervous system. Under hypoxic conditions, the expression of ncam1 in the NEC1 of the gills was inhibited, and the expression level was restored after 6 h of oxygen supply (Figure S1). In addition, the expression levels of two gating genes, calcium voltage-gated channel subunit alpha1 b (cacna1b) and potassium voltage-gated channel subfamily c member 1 (kcnc1), were significantly downregulated after hypoxia. The protein encoded by *cacna1b* is the pore-forming subunit of the N-type voltage-dependent calcium channel (Cav2.2), which controls neurotransmitter release from neurons [37]. *Kcnc1* encodes the α subunit of the voltage-gated potassium channel (Kv3.1), the core function of which is to mediate potassium efflux; it is primarily responsible for high-frequency firing of neurons and the rapid repolarization of action potentials [38]. A similar phenomenon of hypoxia-induced downregulation of voltage-gated calcium channels was found in rat aortic smooth muscle cells (A7r5) [39], which may be a strategy to avoid calcium overload and achieve neuroprotection by reducing calcium influx. Hypoxia inhibits the activity of Kv3.1b channels in L929 cells, prolongs the duration of action potentials, and weakens the ability of neurons to respond to high-frequency stimuli, thereby adapting the activity patterns of neurons to changes in the local oxygen environment [40]. However, these findings are all based on transcriptome level analysis, and further research is still needed to verify them. In NEC2, synaptotagmin-1 (Syt1) is a key synaptic vesicle protein in the nervous system that plays a central role in the calcium-dependent regulation of neurotransmitter release [41]. Pan et al. [21] also detected the expression of *syt1* in zebrafish NECs. Research has shown that 6 days of chronic hypoxia can lead to a decrease in the level of *syt1* protein in the Lymnaea stagnalis ganglion, accompanied by a significant increase in the expression of heat shock protein 70 (hsp70) [42], which is consistent with our results. Fei et al. [42] also confirmed that hsp70 has a protective effect in maintaining presynaptic protein stability. In NECs, 5-HT is one of the main excitatory neurotransmitters. Hypoxia stimulation can cause depolarization and Ca^2+^ influx in NECs, leading to the release of 5-HT [43]. 5-hydroxytryptamine receptor 1B (5-ht1b receptor, htr1b), *htr1b*, belonging to G protein-coupled receptors, can regulate the release of 5-HT, thereby inhibiting neuronal activity and reducing the release of neurotransmitters [44]. 5-ht1b/d antagonists can reduce anxiety-like behavior in zebrafish in new environments [45]. Reed et al. [46] found that there is an independent 5-HT hypoxic signaling pathway in zebrafish gills. S-type NECs release 5-HT, which directly acts on 5-HT3 receptors on the vagus nerve sensory ganglia, mediating respiratory reflex. Therefore, we infer that hypoxia leads to the downregulation of NEC2 *htr1b* levels, which may be an adaptive regulation to enhance the regulation of respiratory reflex. In NEC3, we observed significant downregulation of *slc6a4* during both hypoxia and reoxygenation, indicating that hypoxia suppresses 5-HT uptake by gill NECs in *T. vachellii* (Figure S1). In mammals, *slc6a4* silencing has been demonstrated to reverse apoptosis in PC12 cells. However, further research is needed to determine whether the expression of *slc6a4* on NECs in the gills of *T. vachellii* is related to cell apoptosis. Brain-derived neurotrophic factor (bdnf), one of the most potent neurotrophic factors in the central nervous system, plays a critical role in neuronal survival, differentiation, and synaptic plasticity [47,48]. Our results showed that its transcription level was significantly upregulated after hypoxia. Hypoxia is accompanied by an explosion of reactive oxygen species, and an increase in *bdnf* expression may be a way for cells to cope with this oxidative stress [49]. Notably, our results showed that the expression level of *slc30a7* significantly increased after hypoxia. Slc30a7 has been identified as a potential functional gene contributing to high-altitude hypoxia adaptation in Tibetan pigs [50], but further research is needed in fish.

Transcription factors are important regulatory elements in the transcriptional regulation process [51]. Bhlhe40 is a member of the basic helix–loop–helix family, which can inhibit cell proliferation in a fate-dependent manner under hypoxic conditions [52] (Figure 4). Under hypoxic conditions, the expression of *bhlhe40* and *klf4* involved in promoting G1/S phase arrest in neural stem cells is upregulated [53], confirming the function of *klf4* in inducing growth arrest. In Treg cells, *irf1* has been shown to be a pioneer factor in hypoxia-induced chromatin remodeling [54]. These studies may support the phenomenon of higher activity frequency of these transcription factors in the gill NECs of *T. vachellii* after hypoxia compared to the control group.

In terms of cellular communication, our CellChat-based analysis predicted that under normoxic conditions, the ligand–receptor interactions between NECs and MCs via the NCAM signaling pathway were largely silent (Figure 5C). Following hypoxia, however, the predicted interaction probability between NEC2 and MC2/MC4 increased, suggesting a potential hypoxia-induced activation of intercellular crosstalk between NECs and MCs (Figure 6C). Nevertheless, as CellChat infers potential interactions based on expression profiles rather than directly measuring signaling activity, these predictions should be interpreted as correlative. Further functional experiments are required to determine whether this observed increase is specifically linked to the hypoxia-induced changes in neural-related pathways identified by GSEA in MCs. In addition, the role of notch signaling in goblet cell metaplasia and excessive mucus production remains controversial [55]. Therefore, based on our current research results, the phenomenon of a complete downregulation of the probability of intercellular communication between gill NECs and MCs through the notch signaling pathway remains unexplained. Nevertheless, the inhibitory effect of hypoxia on cellular communication through this signaling pathway has been clearly established.

Erythrocytes in fish gills mainly undertake the task of gas exchange, which occurs between blood and flowing water in the gill capillaries [56]. There are studies showing that fish may enhance their oxygen transport capacity by inducing the proliferation of erythrocytes in peripheral blood and increasing the number of red blood cells to compensate for the slow oxygen transport after continuous hypoxia exposure [57,58]. However, our results show that although there is an upward trend in the number of erythrocytes, there is no significant difference. This may be caused by short-term acute stress. Teleost fish erythrocytes also have certain immune functions [56]. The invariant chain (cd74) mediates the assembly and subcellular traffic of MHC II complexes, which play an essential role in antigen presentation [59]. The downregulation of *cd74* levels in erythrocytes also indicates that hypoxia may inhibit the immune assistance or antigen presentation functions of these cells. In addition, we observed that hypoxia can upregulate the expression of ferritin heavy chain gene (fth1a) in erythrocytes, which can participate in ferroptosis [60,61]. However, whether erythrocytes bind more free Fe^2+^ by upregulating *fth1a* levels and compensatory protection of gill tissue still needs further research.

PVCs have a relatively abundant quantity in the gill epithelial tissue of fish, and they participate in the construction of gas exchange barriers, providing a key interface for the diffusion of oxygen from water to blood [62]. Tight junction protein 2 (tjp2) is one of the constituent proteins of tight junctions, and an essential scaffold protein for tight junction assembly and maintenance [63,64]. It regulates cell bypass permeability by recruiting closing proteins and small-loop closing proteins to the intercellular junctions. Research has shown that the loss of tjp2 function can lead to the breakdown of tight junction structures, which in turn can cause barrier dysfunction [65,66]. Tjp2 regulates the morphology and connectivity stability of epithelial cells by interacting with the actin cytoskeleton. LIM domain and actin binding 1 (lima1) is a cytoskeletal-associated actin binding protein that participates in cytoskeletal regulation and dynamics by inhibiting actin filament depolymerization and cross-linking [67,68]. Our results indicate that after hypoxia, gill PVCs may enhance tight junction structures and maintain cellular cytoskeleton homeostasis by upregulating the levels of *tjp2* and *lima1*.

In *Danio rerio* (Hamilton, 1822), two distinct tuft cell subtypes (TCs I and TCs II) have been identified in gill tissues [21], whereas TCs in *Acipenser dabryanus* (Duméril, 1869) have been detected in intestinal and renal tissues [16]. In the present study, we identified only a single tuft cell population in the gills of *T. vachellii*. TCs are rare chemosensory epithelial cells that monitor environmental stimuli and transmit signals to surrounding tissues through the secretion of neuromodulatory and immunomodulatory molecules [69,70], while also possessing immunological functions. Currently, investigations into gill-resident TCs in fish species remain limited. Our research supports the immune function in gill tissue; for instance, *nlrc3*, *mif*, *cd80*, and *nfkbia* were all significantly changed following hypoxic exposure (Figure 4E). Nevertheless, the functional significance of TCs in gill tissue requires further validation.

Hypoxia stress can directly lead to tissue damage in fish. A study in *Eleutheronema tetradecylum* (Shaw, 1804) showed that in an environment with DO of 2 mg/L for 2 h, the epithelial cells of the gill tissue were elongated as a whole, and some epithelial cells were swollen, protruding, and even ruptured [71]. *Ctenopharyngodon idellus* (Valenciennes, 1844) exhibited necrosis of gill epithelial cells after 96 h of hypoxia (DO: 1 mg/L), and electron microscopy results showed significant swelling of the endoplasmic reticulum in the gills [72]. After acute hypoxia for 6 h (DO: 1.55 mg/L), the mitochondrial volume in the MRCs of *Trachinotus ovatus* (Linnaeus, 1758) increased and appeared circular [73]. Yellow catfish is a scaleless fish, which means that its skin is more susceptible to damage in the absence of oxygen compared to scaled fish. The study of Wang et al. [74] showed that *P. fulvidraco* had enlarged skin mucus cells after 6 h of hypoxia (DO: 1.0 mg/L), and long-term hypoxia (96 h) will cause the epithelial layer to thin out. After hypoxia (DO: 1 mg/L), the number of epithelial cells in the skin of *A. dabryanus* increased, while the number of MCs decreased [25]. Acute hypoxia does indeed cause changes in the skin structure of *T. vachellii* and timely recovery can also alleviate the damage (Figure 7 and Figure 8). The muscle fibers of A. dabryanus showed degeneration and contained a small amount of vacuoles after hypoxia [25]. Our research results also confirm that hypoxia can cause damage to fish muscles. Overall, the above results not only confirm that hypoxia can cause damage to tissues but also indicate that restoring oxygen supply over a period of time can alleviate these damage phenomena, which guides us to develop recovery plans after acute hypoxia.

A limitation of this study is that the control group was collected at baseline, while hypoxia and recovery groups were collected at later time points. Thus, some transcriptional changes may reflect time-dependent effects, handling stress, or circadian variation rather than hypoxia alone. As we lack time-matched normoxic controls, these confounding factors cannot be fully excluded. Our results should therefore be interpreted with caution, and future studies with time-matched controls are needed to validate the hypoxia-specific effects.

## 5. Conclusions

Overall, in this study, we established an acute hypoxia–reoxygenation model in *T. vachellii* and generated a comprehensive gill single-cell transcriptomic atlas to investigate the cellular and molecular mechanisms underlying hypoxia responses (Figure 9). The three clusters of NECs we detected seem to have different functions. Hypoxia may inhibit cell growth by upregulating the frequency of activity of corresponding transcription factors. We speculate that MCs may be regulated by non-neuronal neurotransmitter signals (GABA; 5-hydroxytryptamine pathway) under hypoxic conditions, but further research is needed to confirm this. Erythrocyte proliferation does not seem to be affected by acute hypoxia, but hypoxia may affect the regulation of PVC stability. In addition, TCs, as “chemical receptor” cells—which have received much attention in recent years—have been identified in our research and suspected to have immune regulatory functions under hypoxic stress, providing new ideas for our future research on fish hypoxia immune regulation. Based on our observation of the organizational structure, we have also determined that timely restoration of support can effectively alleviate the damage caused by hypoxia stress. These results provide single-cell atlas and data support for the acute hypoxia and reoxygenation model of the gill, deepen our understanding of the mechanisms of hypoxia adaptation in aquatic animals, and provide effective theoretical support for us to develop strategies to cope with hypoxia.

## Figures and Tables

**Figure 1:**
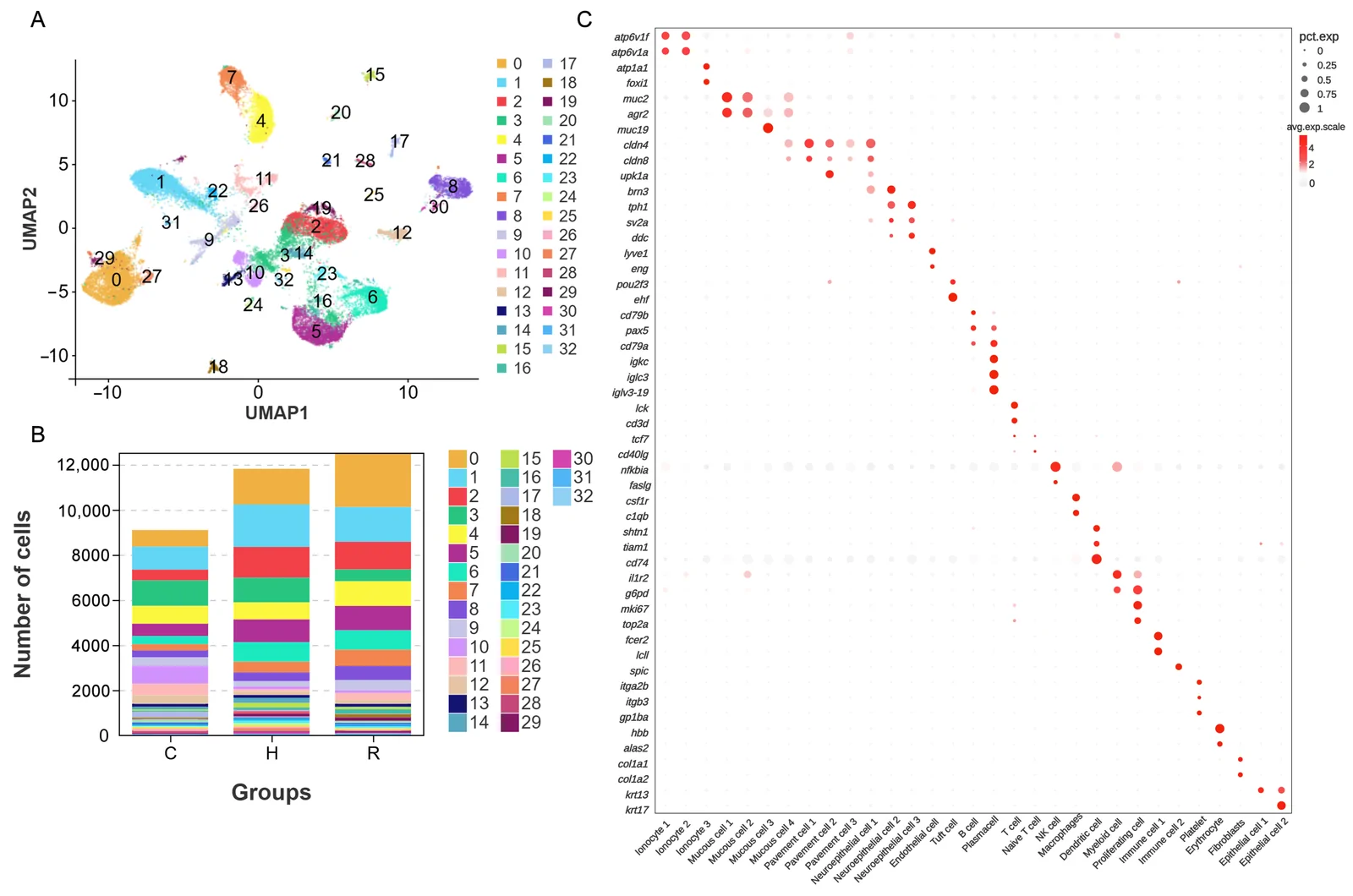
Overview of single-cell sequencing of gill tissue in *T. vachellii.* (**A**) UMAP plot of cell subpopulation classification in gill tissue of *T. vachellii*; (**B**) stacking diagram of cell subpopulations in each group (*x*-axis: marker gene; *y*-axis: cell subpopulation); (**C**) bubble plot of the main marker genes for identified cell clusters (different colors indicate different cell types, the height of each colored segment represents the number of cells of that cell type).

**Figure 2:**
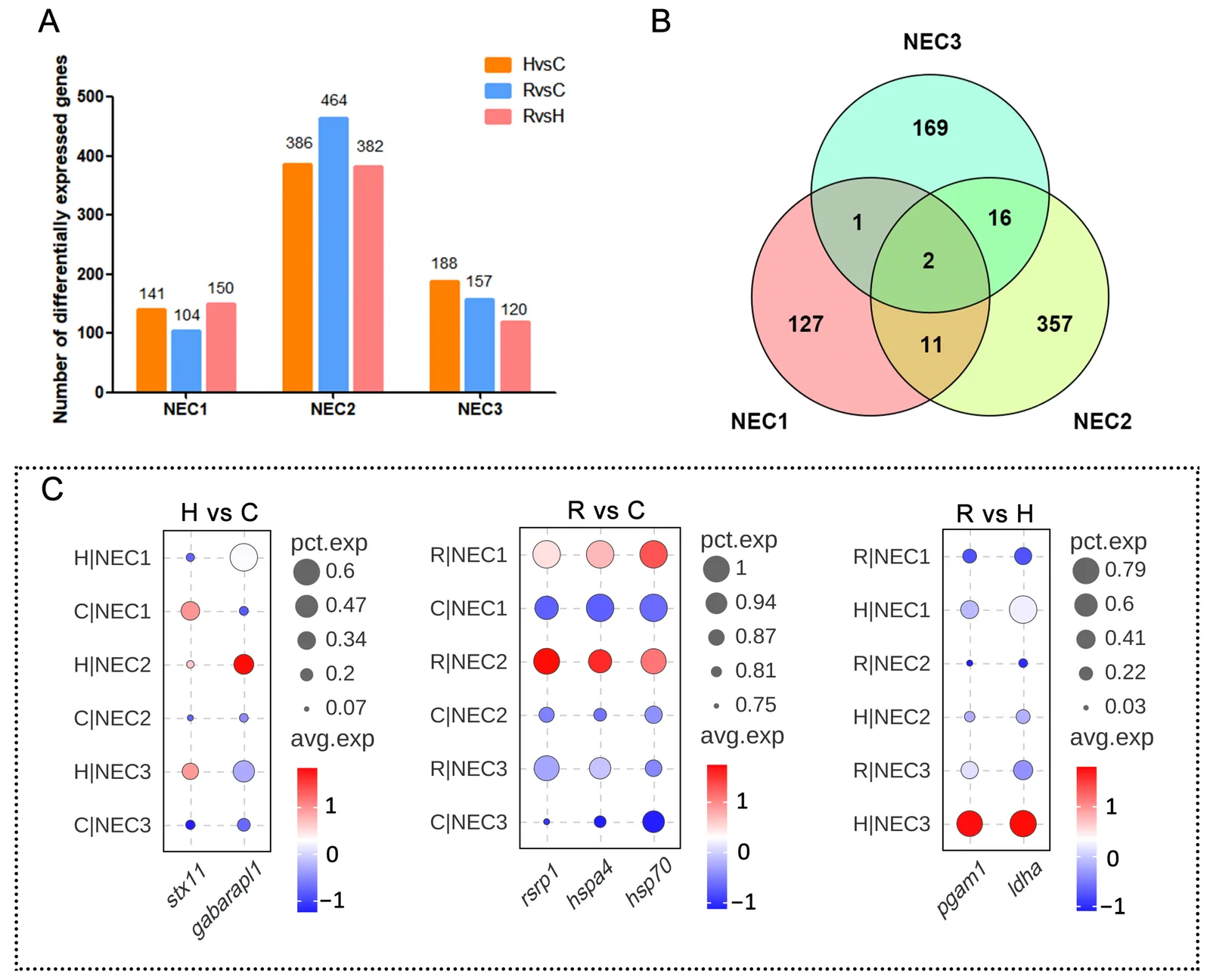
Analysis of differential gene expression in NECs after hypoxia and reoxygenation. (**A**) Bar chart of DEGs in various NECs after hypoxia and reoxygenation; (**B**) Wayne diagram of DEGs in various NECs after hypoxia (HvsC); (**C**) bubble plot of common DEGs among various NECs after hypoxia and reoxygenation.

**Figure 3:**
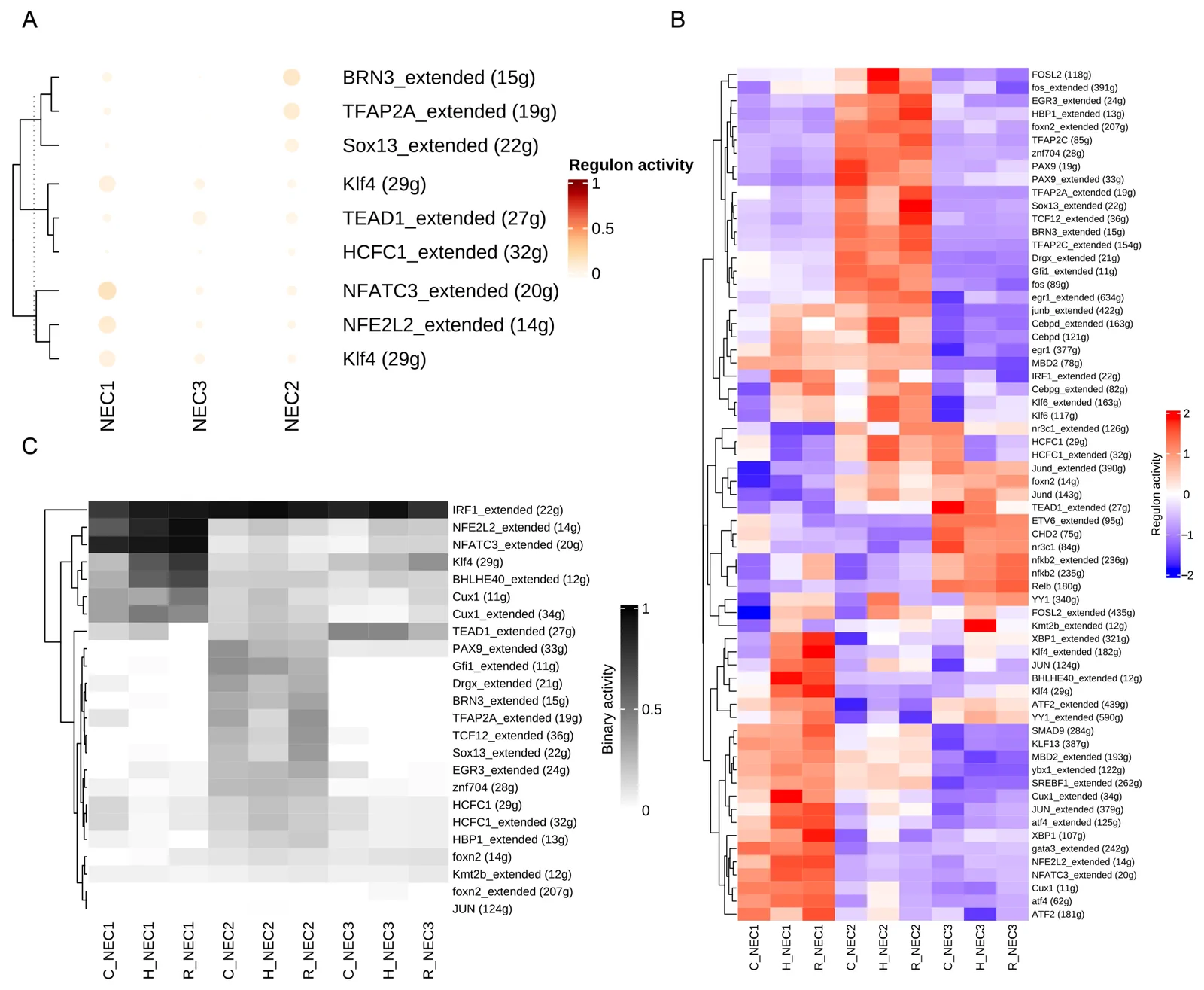
Transcription factor analysis of NECs. (**A**) Bubble chart of the top 3 differentially enriched regulons identified among NECs; (**B**) the heatmaps of mean regulon activity among NECs; (**C**) the heatmaps of mean binary activation among NECs.

**Figure 4:**
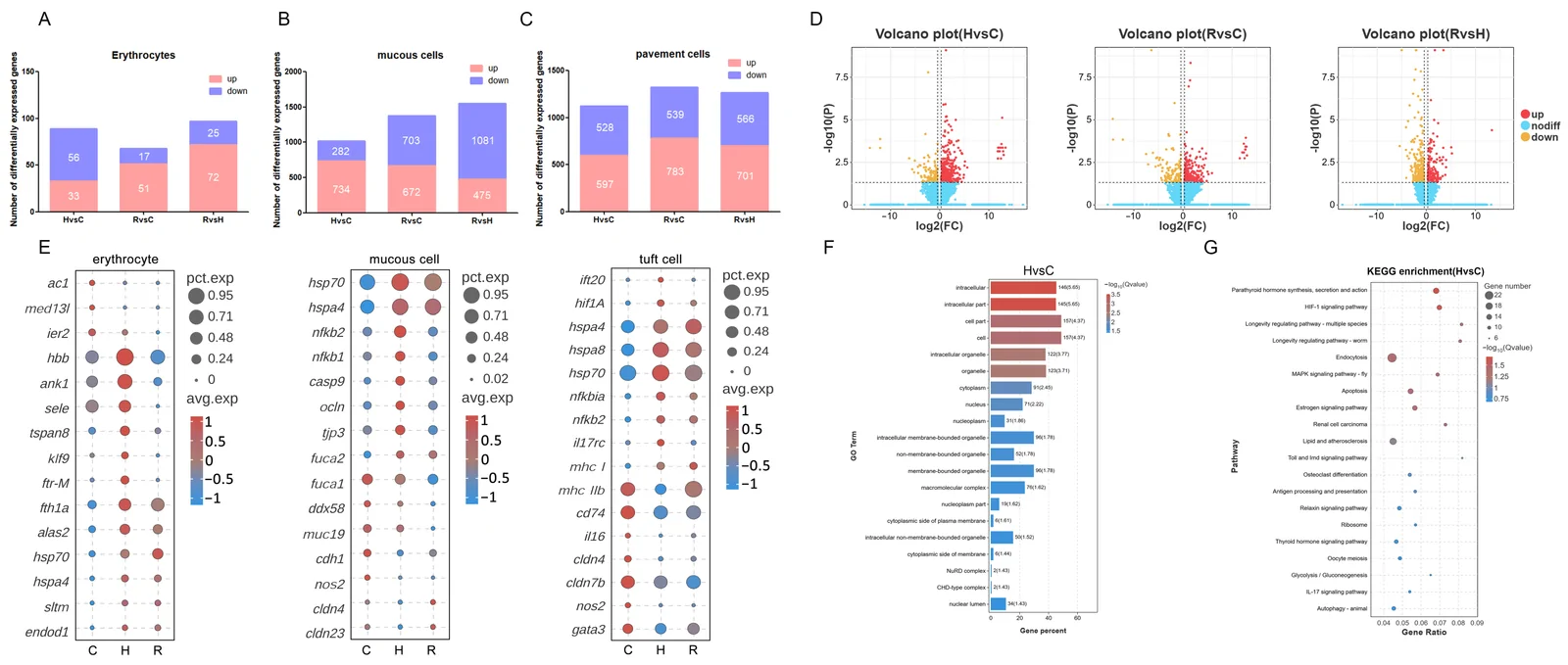
Differential analysis of cells after hypoxia and reoxygenation. (**A**–**C**) Bar chart of differential gene expression in different cells after hypoxia and reoxygenation; (**D**) volcanic map of differential gene expression in TCs after hypoxia and reoxygenation; (**E**) bubble chart of partial DEGs in erythrocyte, MC, PVC and TC (HvsC); (**F**) GO enrichment map of DEGs in TCs after hypoxia; (**G**) KEGG enrichment signaling pathway of DEGs in TCs after hypoxia.

**Figure 5:**
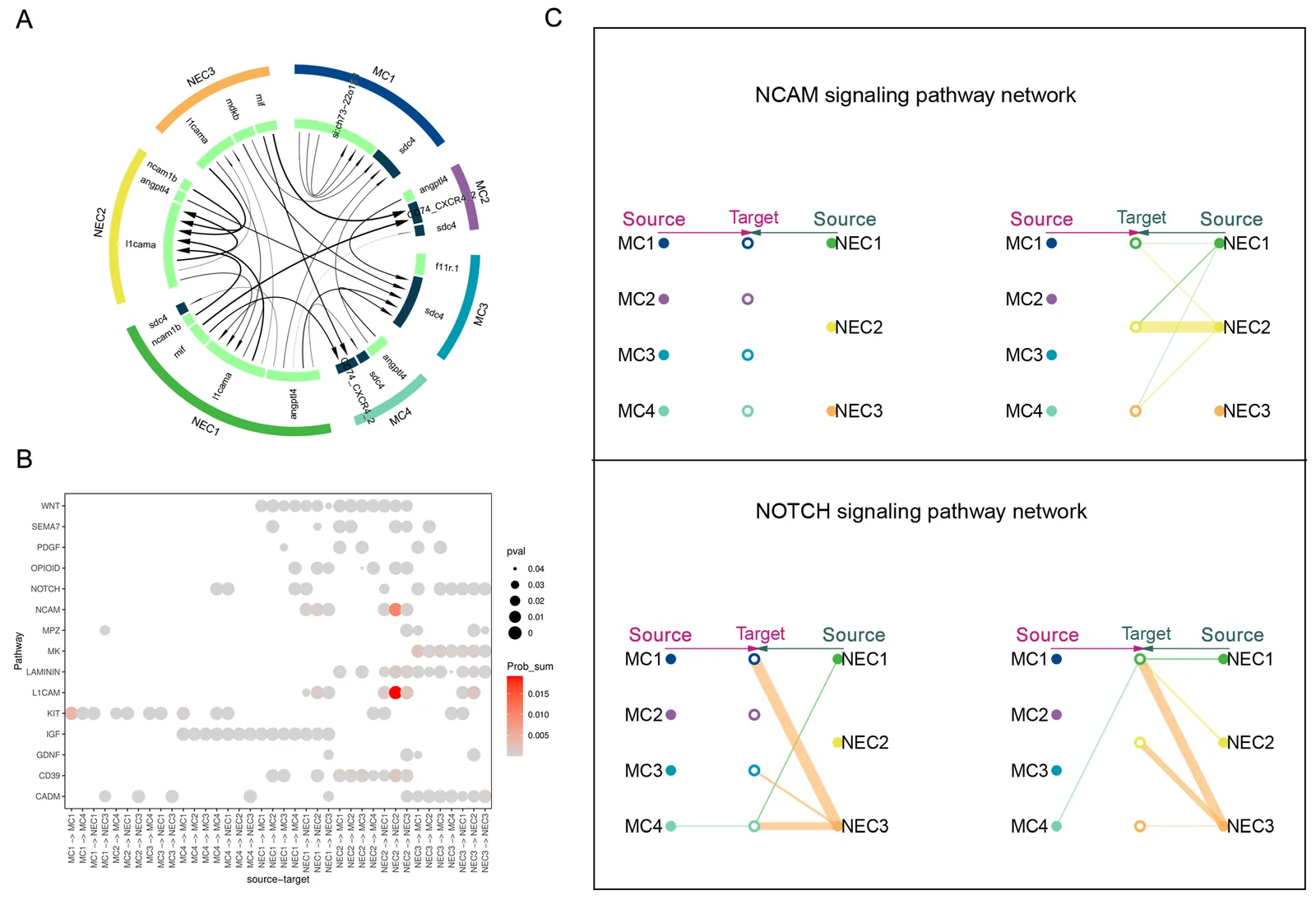
Overview of cell communication between NECs and MCs. (**A**) Network diagram of ligands and receptors. In the figure, we show the top 30 rows of ligand–receptor relationships with significant communication probability (*p*-value ≤ 0.05) and sorted by the product of ligand gene expression and receptor gene expression from high to low; (**B**) probability bubble plot of receptor pairing between cell subpopulations. The size of the bubble represents the *p*-value, and the smaller the *p*-value, the larger the bubble. The color represents the magnitude of the probability, the larger the color, the darker the color. Each cluster of cells only displays the top 5; (**C**) cell communication hierarchy diagram of signaling pathway. In the left figure, the left and right columns represent ligand cells, while the middle column represents receptor cells. The line connecting the two columns represents the communication relationship between each ligand cell and the receptor cell. The color of the line is consistent with that of the ligand cell, and the thickness indicates the strength of communication. The stronger the line, the thicker the line. In the middle column of the image on the right, the cells in the right column are used as receptor cells, while the rest are the same as in the left image.

**Figure 6:**
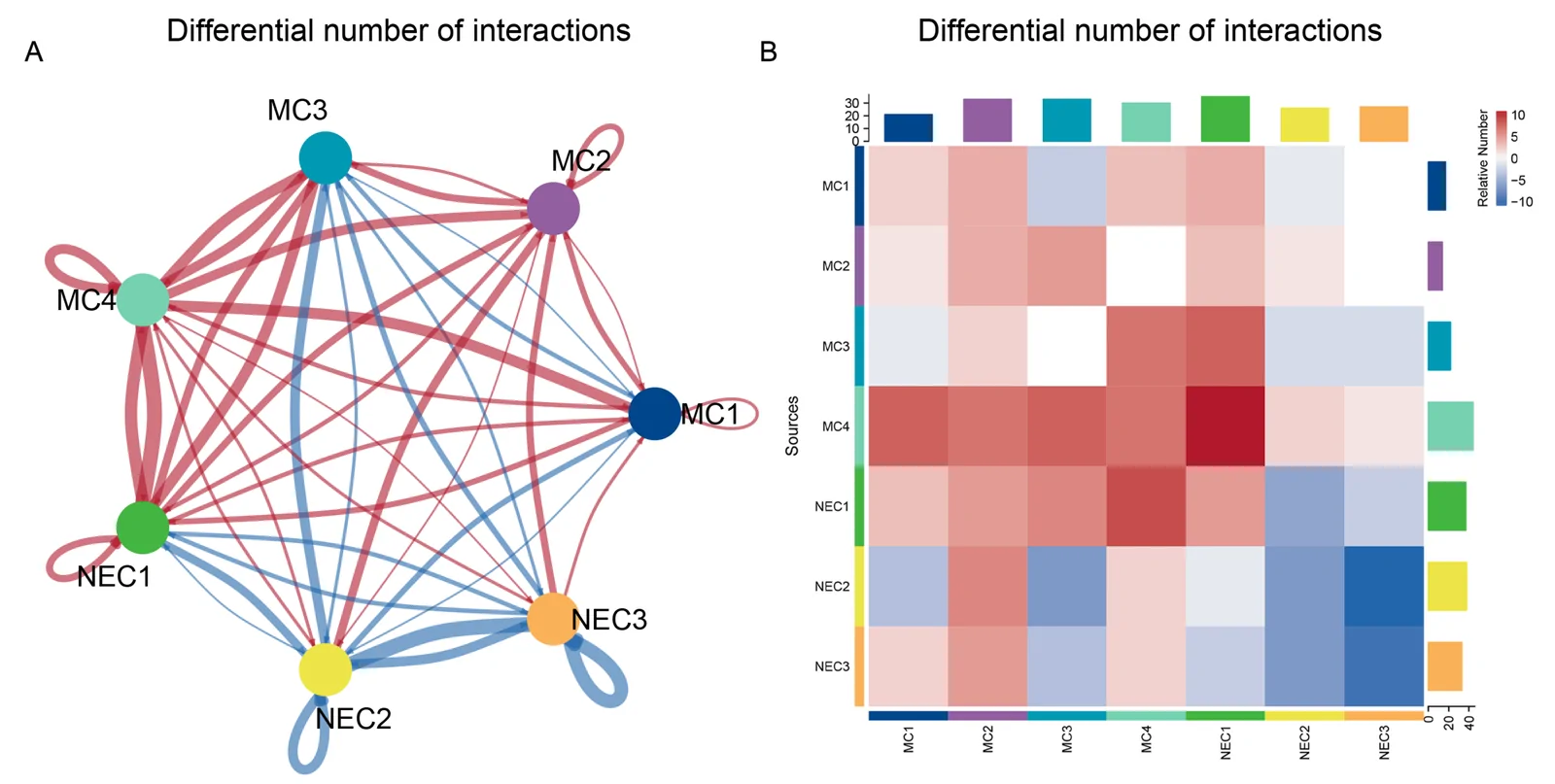

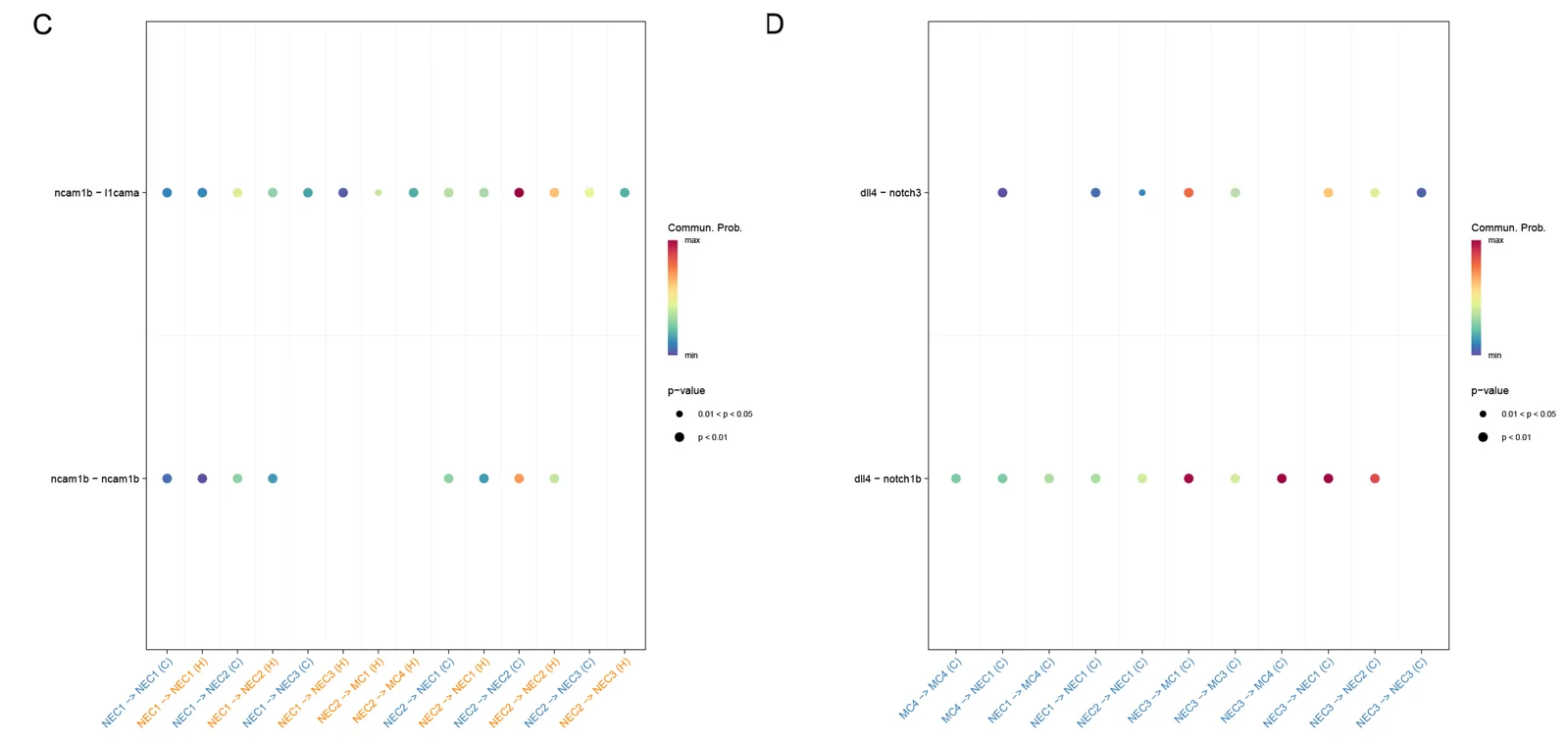
Comparison of cellular communication differences between NECs and MCs after hypoxia (HvsC). (**A**) Cell communication differential network diagram. The different colors of the outer circle indicate different cell subgroups, and the size indicates the number of receptor pairs in each subgroup. The larger the circle, the higher the ratio of receptor pairs between cells. The blue line indicates strong communication in the control group, while the red line indicates strong communication in the comparison group. The thicker the line, the stronger the degree of communication change. (**B**) Cell communication differential heatmap. The vertical axis represents ligand cells, and the horizontal axis represents receptor cells. The color of the heatmap represents the difference in the number of ligand–receptor pairs between cells, and the darker the color, the stronger the communication. The upper and right columns represent the cumulative difference in the number of receptor pairs along the vertical and horizontal axes. (**C**) Probability difference bubble plot of NCAM signaling pathway ligand–receptor pairs. The horizontal axis represents cell pairs, distinguishing samples by color. The vertical axis represents the ligand–receptor pairs. The size of the bubble represents the *p*-value, and the smaller the *p*-value, the larger the bubble. The color represents the probability of communication. (**D**) Probability difference bubble plot of NOTCH signaling pathway ligand–receptor pairs.

**Figure 7:**
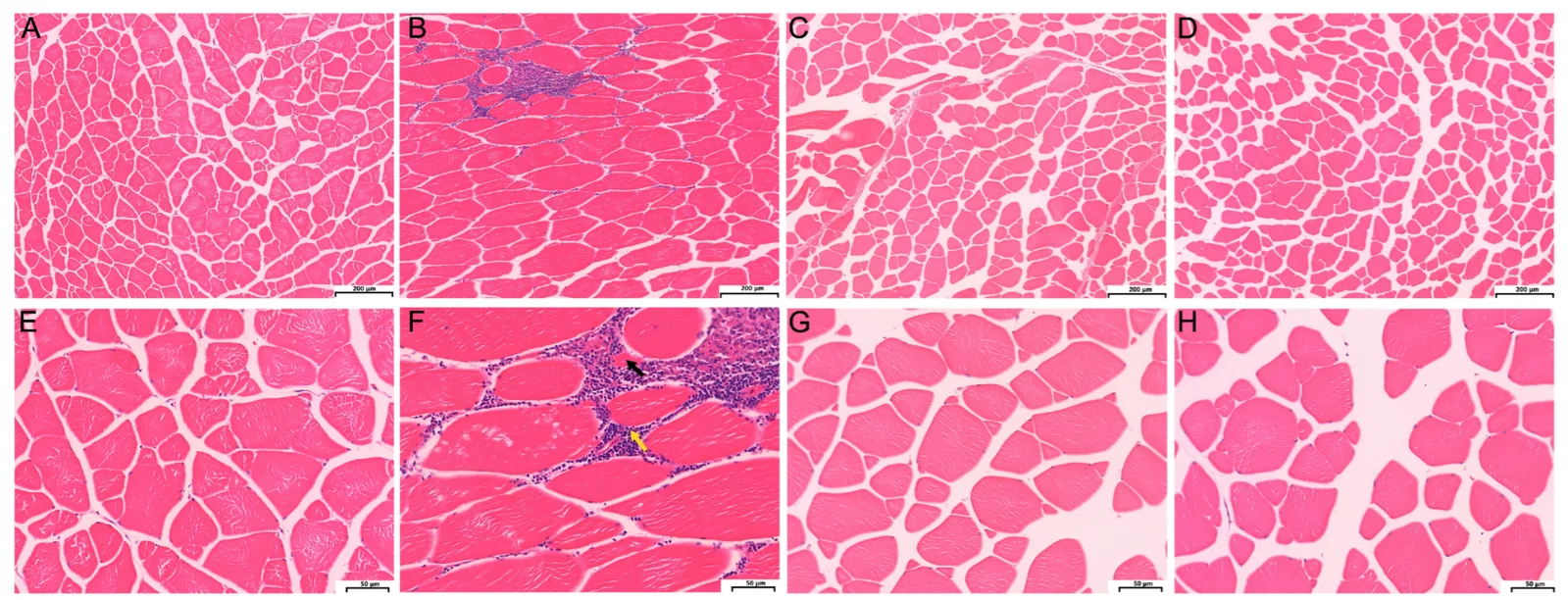

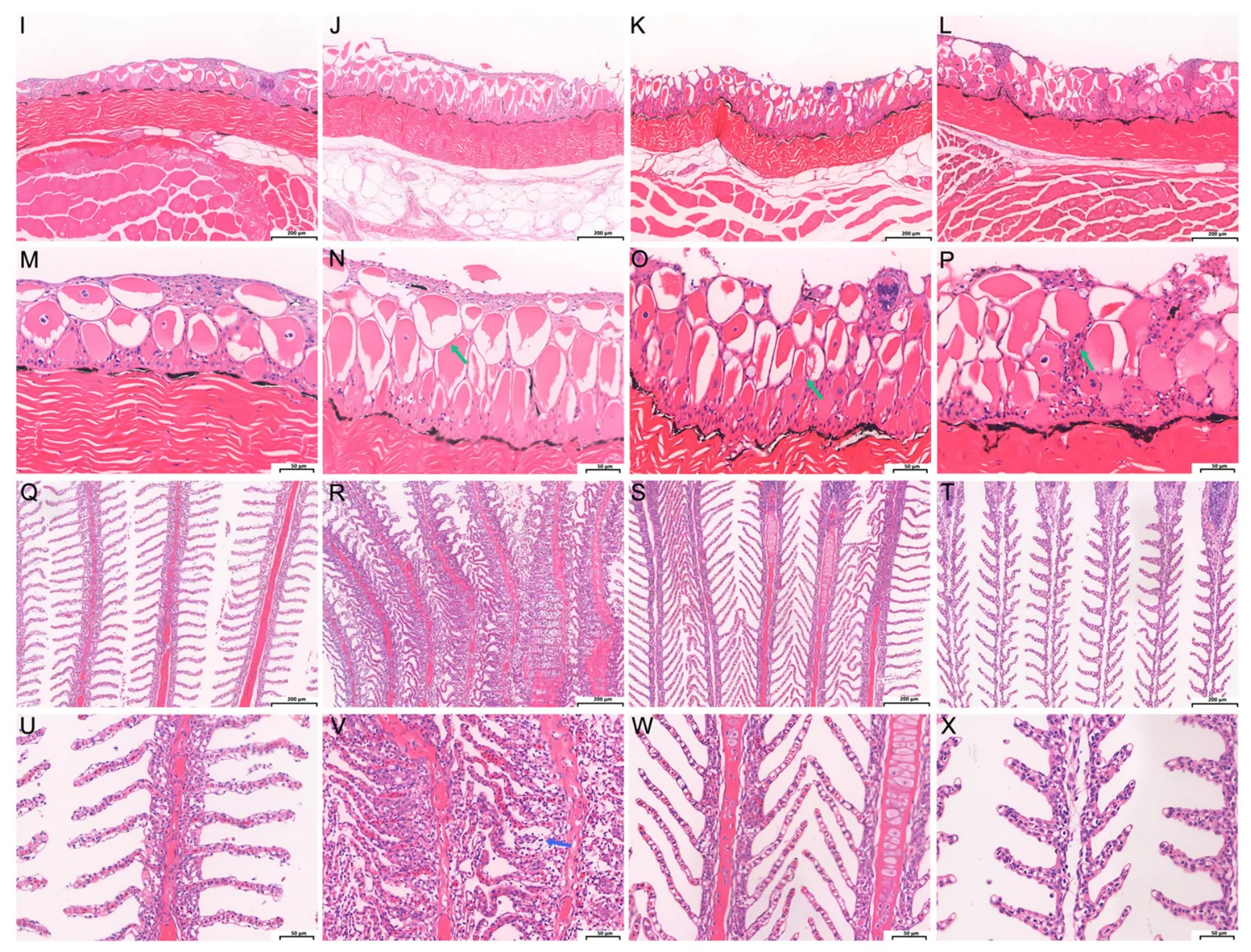
H&E staining sections. (**A**–**H**) muscle, (**I**–**P**) skin, (**Q**–**X**) gill; (**A**–**D**,**I**–**L**,**Q**–**T**) × 100; (**E**–**H**,**M**–**P**,**U**–**X**) × 400; (**A**,**E**,**I**,**M**,**Q**,**U**) control group; (**B**,**F**,**J**,**N**,**R**,**V**) hypoxia group; (**C**,**G**,**K**,**O**,**S**,**W**) recovery 6 h; (**D**,**H**,**L**,**P**,**T**,**X**) recovery 24 h; The arrow: black refers to muscle fiber necrosis; yellow represents lymphocytes; green indicates thickening of the epidermal layer of the skin; blue indicates epithelial cell necrosis.

**Figure 8:**
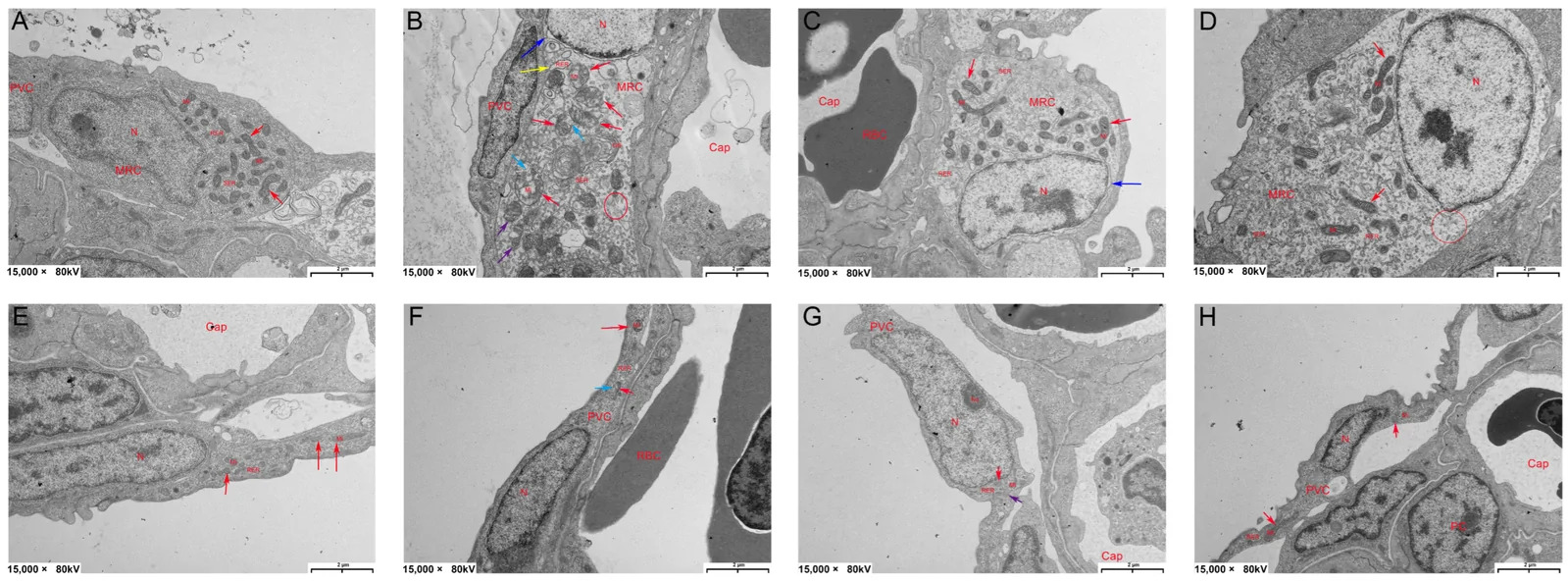
Transmission electron micrographs. (**A**–**H**) 15,000×, 80 kV; (**A**–**D**) MRC; (**E**–**H**) PVC; (**A**,**E**) control group; (**B**,**F**) hypoxia group; (**C**,**G**) recovery 6 h; (**D**,**H**) recovery 24 h; the red arrow refers to mitochondria with normal morphological structure. Widening of perinuclear space with dark blue arrow, swelling of mitochondria with red arrow, mild swelling of mitochondria with purple arrow, rupture of outer membrane of mitochondria with light blue arrow, expansion of rough endoplasmic reticulum with yellow arrow, and loss of ribosomes with red circle; PVC: pavement cells, MRC: mitochondria-rich cells, RER: rough endoplasmic reticulum, SER: smooth endoplasmic reticulum, N: nucleus, Mi: mitochondrion, GB: golgi apparatus; Cap: Capillary; No: nucleolus.

**Figure 9:**
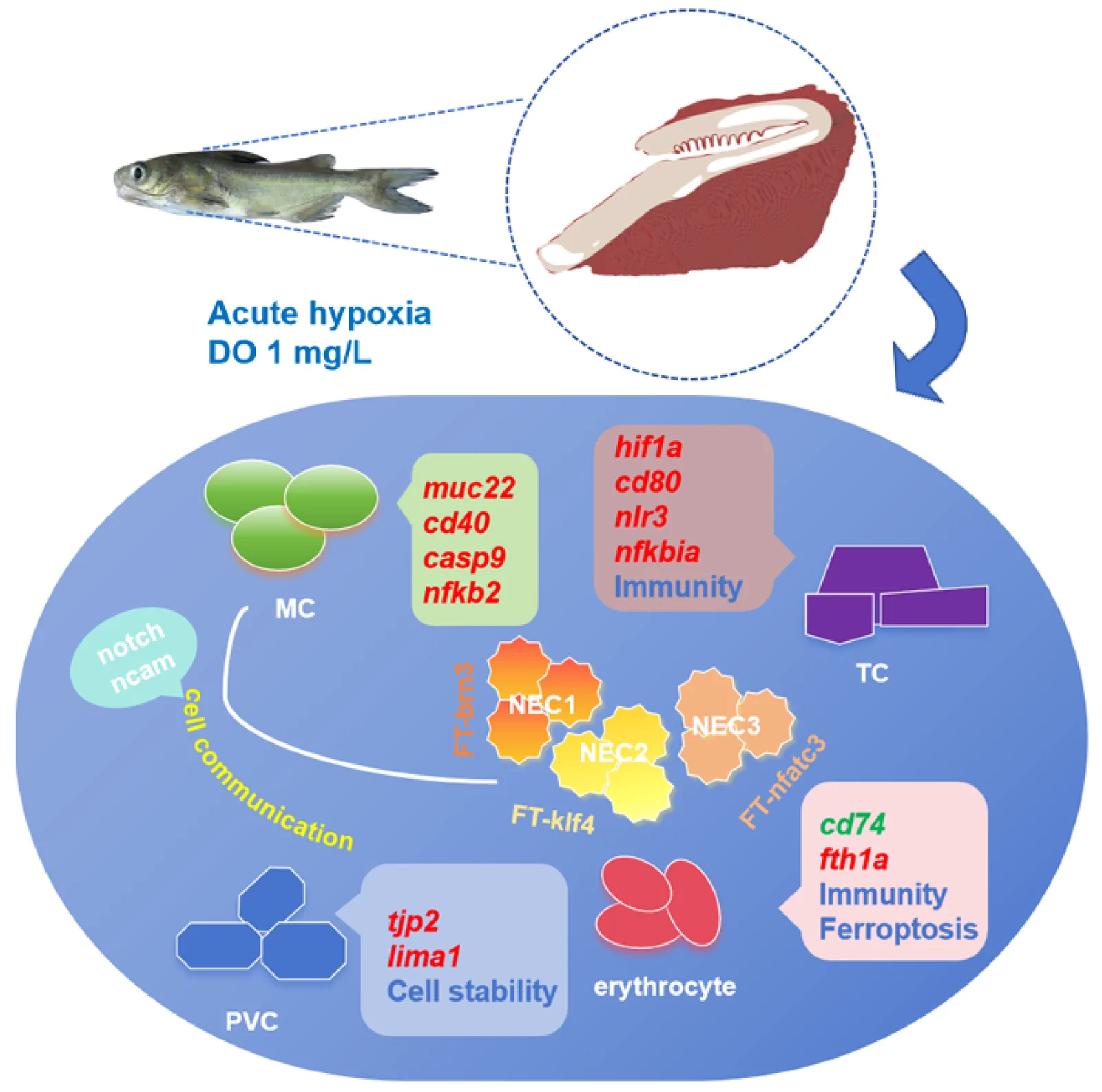
Summary model of *T. vachellii* gill cell response to acute hypoxia and reoxygenation. FT: transcription factor.

## Data Availability

The raw data supporting the conclusions of this article will be made available by the authors on request.

## Supplementary Materials

The following supporting information can be downloaded at https://www.mdpi.com/article/10.3390/cells15191747/s1. Figure S1: Analysis of DEGs among NECs. A: The Venn diagram of DEGs among NECs RvsC; B: The Venn diagram of DEGs among NECs RvsH; C: The bubble chart of partial DEGs among NECs; Figure S2: UMAP prediction of average regulator activity, binary activity and gene expression of *brn3* extended (15 g), *tead1* extended (27 g) and *nfatc3* extended (20 g) in NECs; Figure S3: Analysis of DEGs in gill erythrocyte of *T. vachellii*. A: GO enrichment histogram of DEGs among groups; B: KEGG enrichment bubble plot of DEGs between groups; Figure S4: Analysis of DEGs in gill MCs of *T. vachellii*. A: GO enrichment histogram of DEGs among groups; B: KEGG enrichment bubble plot of DEGs between groups; C: GSEA analysis chart of DEGs between groups. Figure S5: Analysis of DEGs in gill PVCs of *T. vachellii.* A: GO enrichment histogram of DEGs among groups; B: KEGG enrichment bubble plot of DEGs between groups; Figure S6: Analysis of DEGs in gill tuft cells of *T. vachellii*. A: GO enrichment histogram of DEGs among groups; B: KEGG enrichment bubble plot of DEGs between groups; Table S1: List of marker genes for identified cell clusters; Table S2: DEGs among groups for NEC1; Table S3: DEGs among groups for NEC2; Table S4: DEGs among groups for NEC3; Table S5: DEGs among groups for erythrocyte; Table S6: DEGs among groups for MC; Table S7: DEGs among groups for PVC; Table S8: DEGs among groups for TC.

## Abbreviation

Ach	acetylcholine
agr2	anterior gradient 2
alas2	5′-aminolevulinate synthase 2
atp1a1	sodium/potassium-transporting ATPase subunit alpha-1
atp6v1a	V-type proton ATPase catalytic subunit A
atp6v1e1	ATPase H+ transporting V1 subunit E1
atp6v1f	ATPase H+ transporting V1 subunit F
avil	Advillin
bdnf	brain-derived neurotrophic factor
bhlhe40	basic helix–loop–helix family member e40
brn3	pou class 3 homeobox
c1qa	complement component 1, q subcomponent, a chain
c1qb	complement component 1, q subcomponent, b chain
c1qc	complement component 1, q subcomponent, c chain
cacna1b	calcium voltage-gated channel subunit alpha1 b
Cav2.2	pore-forming subunit of the N-type voltage-dependent calcium channel
cd40lg	cd40 ligand
cdh5	cadherin 5
cDNA	complementary DNA
cldn4	claudin 4
cldn8	claudin 8
clec3a	C-type lectin domain family 3 member a
col1a1	collagen type I alpha 1 chain
col1a2	collagen type I alpha 2 chain
col12a1	collagen type XII alpha 1 chain
COVID	Coronavirus Disease 2019
csf1r	Colony-stimulating factor 1 receptor
dcn	decorin
ddh	meso-diaminopimelate-D-dehydrogenase
DEGs	differentially expressed genes
DO	dissolved oxygen
E	erythrocytes
ehf	ETS homologous factor
eng	endoglin
epx	eosinophil peroxidase
fabp4	fatty acid binding protein 4
faslg	fas ligand
fcgbp	fc gamma binding protein
GB	golgi apparatus
GEMs	gel beads in emulations
GO	Gene Ontology
gp1ba	glycoprotein Ib platelet subunit alpha
hbb	hemoglobin subunit beta
hif-1	hypoxia-inducible factor 1
HR cells	H+-ATPase-rich cells
5-HT	5-hydroxytryptamine
htn1	histatin 1
htr1b	5-hydroxytryptamine receptor 1B (5-hr1b receptor)
HUVECs	human umbilical vein endothelial cells
igkc2	immunoglobulin kappa constant 2
iglc3	immunoglobulin lambda constant 3
ighv4–59	immunoglobulin heavy variable 4–59
iglv3–19	immunoglobulin lambda variable 3–19
ilr2	interleukin 2 receptor
irf1	interferon regulatory factor 1
itga2b	integrin subunit alpha 2b
itgb3	integrin subunit beta 3
kcnc1	potassium voltage-gated channel subfamily c member 1
KEGG	Kyoto Encyclopedia of Genes and Genomes
klf4	kruppel like factor 4
krt13	keratin 13
krt17	keratin 17
Kv3.1	kcnc1 encodes the α subunit of the voltage-gated potassium channel
lck	lymphocyte-specific protein tyrosine kinase
lyve5	lymphatic vessel endothelial hyaluronan receptor 5
MC	mucous cells
Mi	mitochondrion
mif	macrophage migration inhibitory factor
MRC	mitochondria-rich cells
muc2	mucin 2
muc5	mucin 5
muc19	mucin 19
NaR cells	Na+/K+-ATPase-rich cells
ncam	neural cell adhesion molecule
ncam1	neural cell adhesion molecule 1
NECs	neuroepithelial cells
nfatc3	nuclear factor of activated T cells 3
nfe2l2	nuclear factor, erythroid 2 like 2
nfkβia	NF-kappa-B inhibitor alpha
NK cells	Natural killer cells
nlrc3	NLR family CARD domain-containing protein 3
No	nucleolus
pax5	paired box 5
PCR	polymerase chain reaction
pecam1	platelet and endothelial cell adhesion molecule 1
pou2f3	pou class 2 homeobox 3
PVC	pavement cells
RER	rough endoplasmic reticulum
scRNA seq	single-cell RNA sequencing
SER	smooth endoplasmic reticulum
slc6a4	serotonin transporter
slc30a7	solute carrier family 30 member 7
spic	Spi-C transcription factor
srgap2	SLIT-ROBO Rho GTPase-activating protein 2
sv2a	synaptic vesicle glycoprotein 2a
TC	tuft cell
tcf7	transcription factor 7
tead1	tea domain transcription factor 1
TEM	transmission electron microscopy
tfrc	transferrin receptor
tiam1	T cell lymphoma invasion and metastasis 1
tox	thymocyte selection associated high mobility group box
tph1	tryptophan hydroxylase 1
t-SNE	t-distributed stochastic neighbor embedding
UMAP	Uniform Manifold Approximation and Projection
UMI	unique molecular identifier
upk1a	uroplakin 1a
znf366	zinc finger protein 366
