# Supplementary material for: Single-Cell Transcriptomic Landscape of the Gill in Hypoxia-Stressed Tachysurus vachellii (Richardson, 1846): Insights into Cell-Type-Specific Responses of Different Epithelial Cells and Erythrocytes
Source: Cells. 2026 Sep 24;15(19):1747. doi: 10.3390/cells15191747 (PMC13664461; doi:10.3390/cells15191747)
Supplement: Supplementary file 1 [file cells-15-01747-s001.zip › cells-4537297 - Supplementary Materials.pdf]

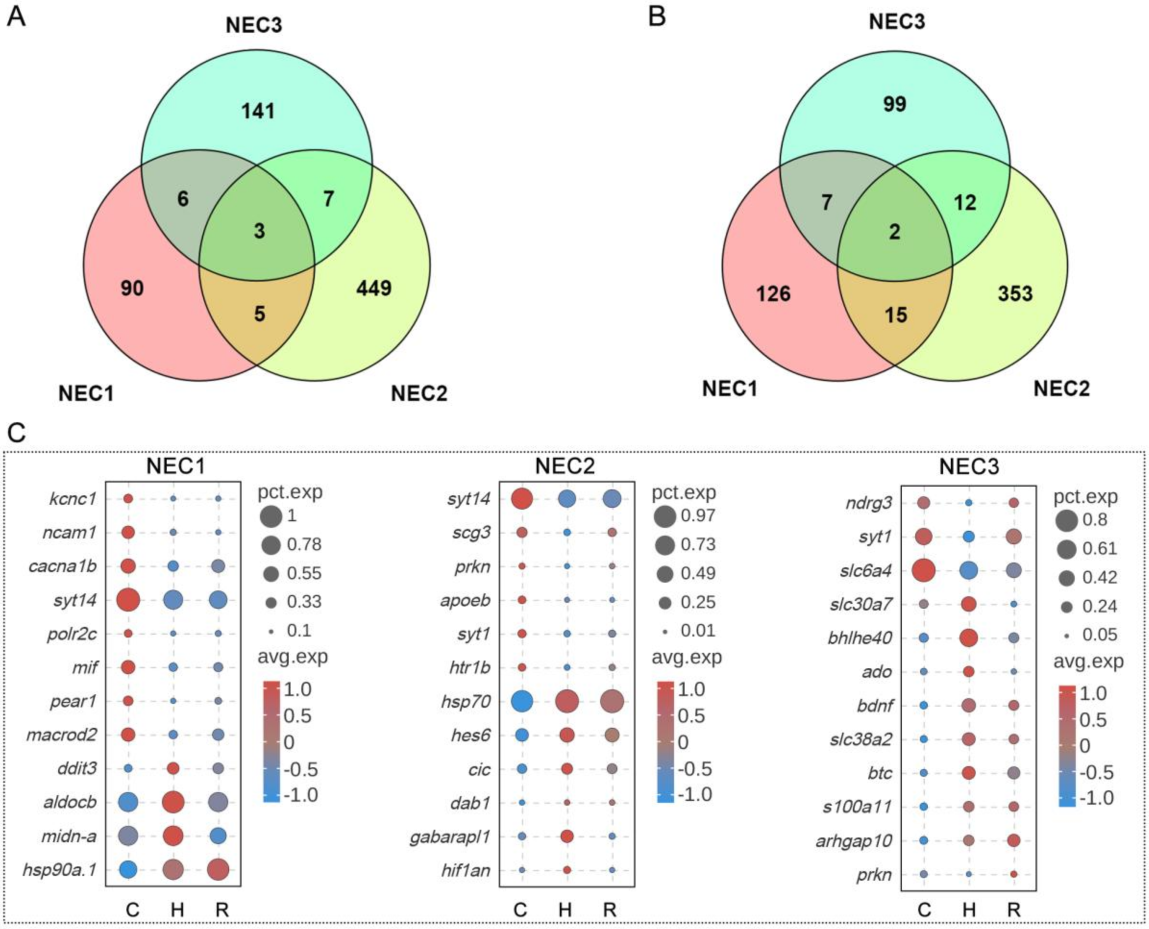

Figure S1. Analysis of DEGs among NECs. A: the Venn diagram of DEGs among NECs RvsC; B: the Venn diagram of DEGs among NECs RvsH; C: the bubble chart of partial DEGs among NECs.

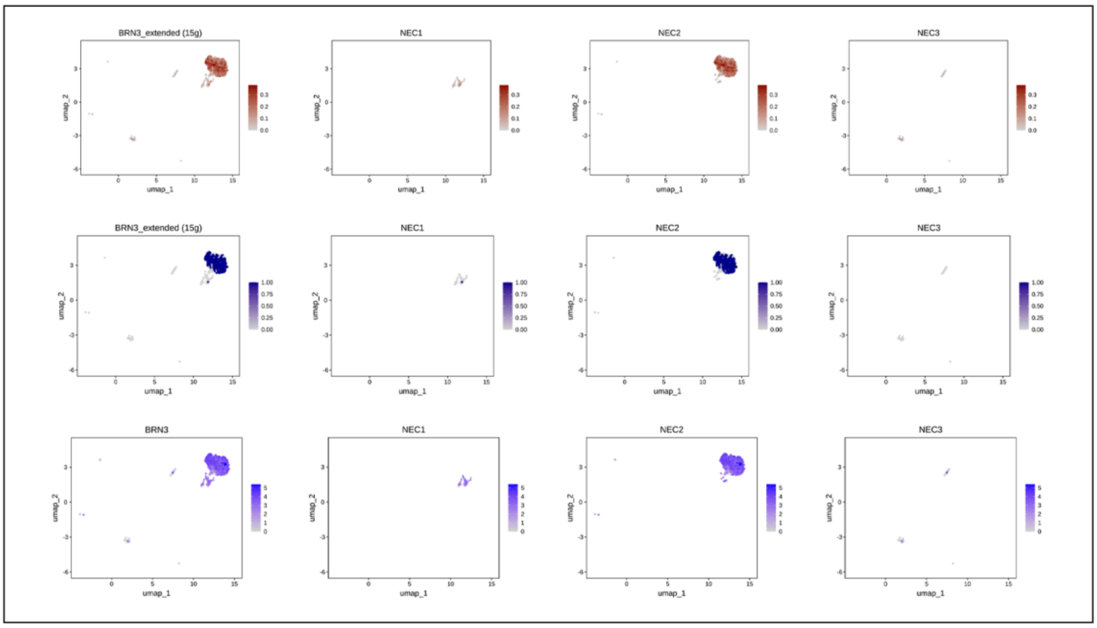
BRN3_extended (15g)
NEC1
NEC2
NEC3
BRN3_extended (15g)
NEC1
NEC2
NEC3
BRN3
NEC1
NEC2
NEC3
umap_1
umap_2

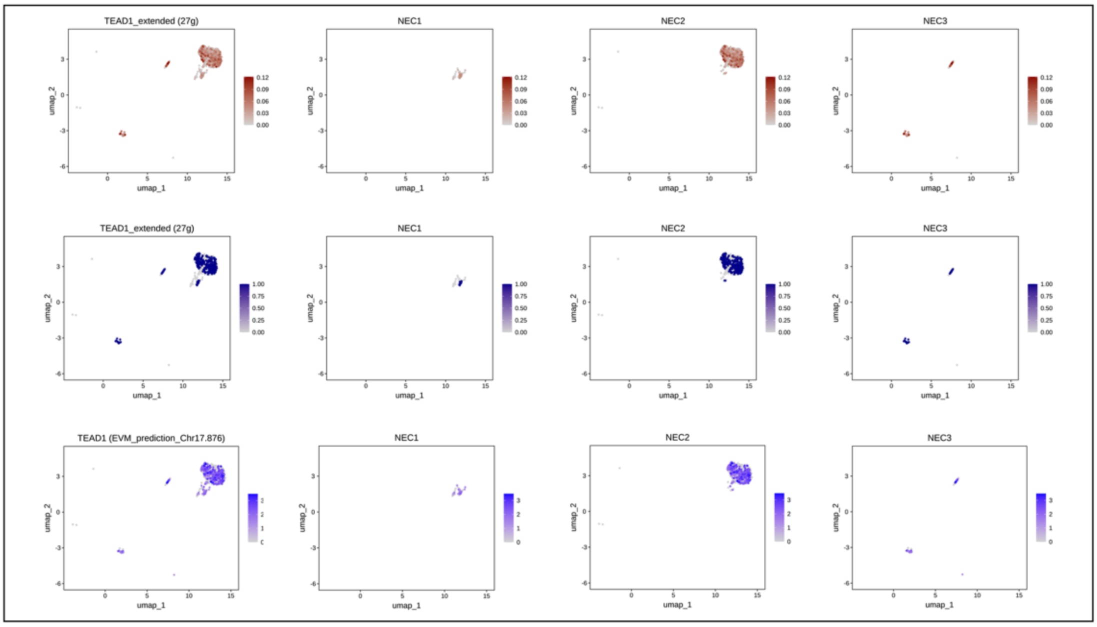
TEAD1_extended (27g)
NEC1
NEC2
NEC3
TEAD1_extended (27g)
NEC1
NEC2
NEC3
TEAD1 (EVM_prediction_Chr17.876)
NEC1
NEC2
NEC3
umap_1
umap_2

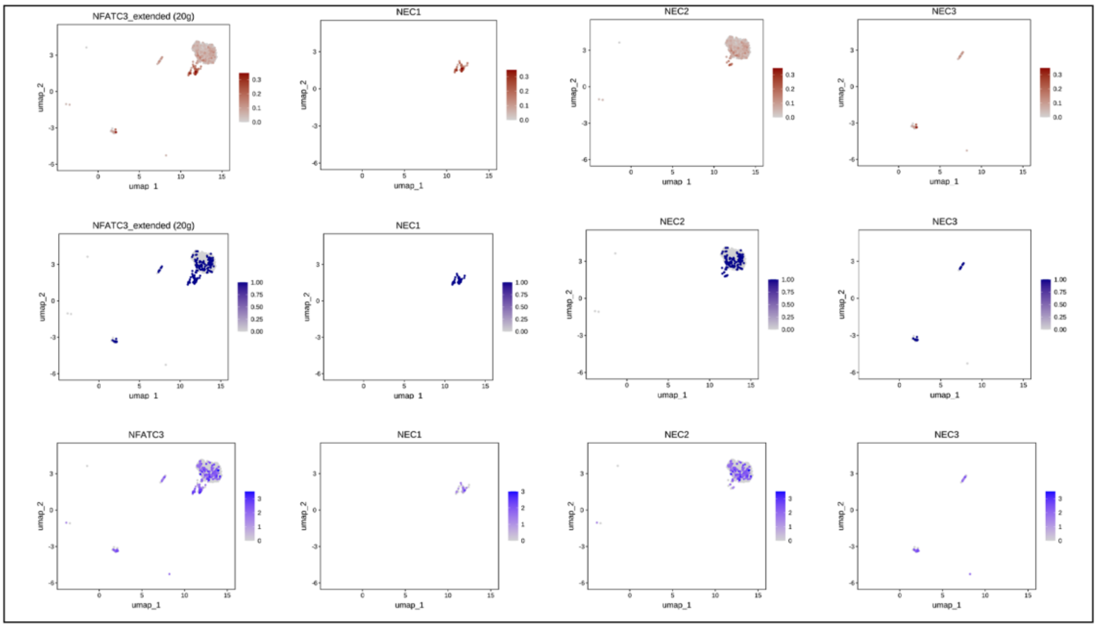
NFATC3_extended (20g)
NEC1
NEC2
NEC3
NFATC3_extended (20g)
NEC1
NEC2
NEC3
NFATC3
NEC1
NEC2
NEC3
umap_1
umap_2

Figure S2. UMAP prediction of average regulator activity, binary activity, and gene expression of *brn3* extended(15g), *tead1* extended(27g), and *nfatc3* extended(20g) in NECs.

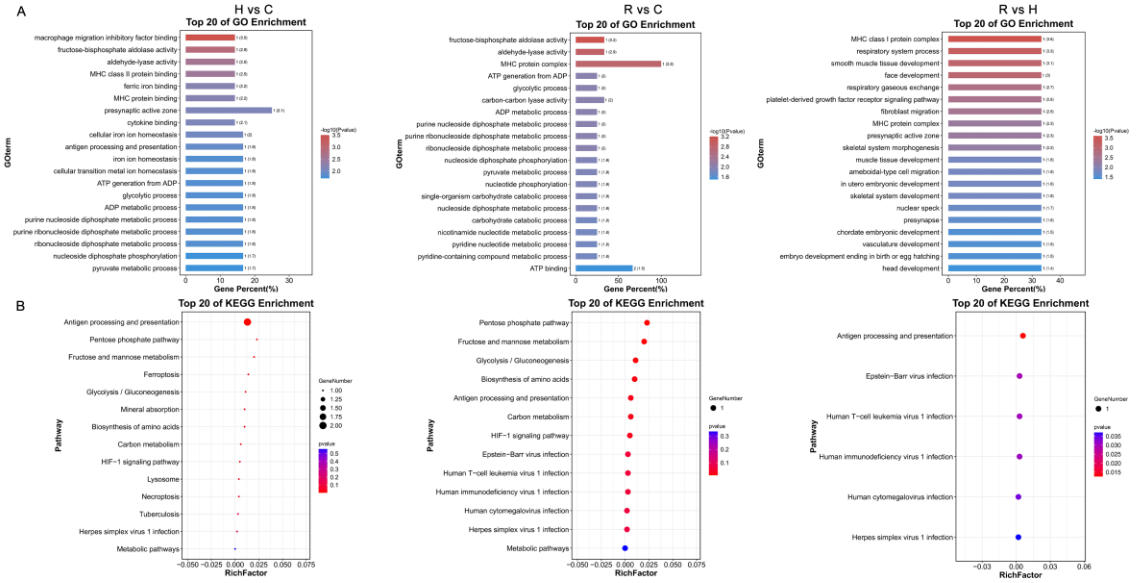

Figure S3. Analysis of DEGs in gill erythrocytes of *T. vachellii*. A: GO enrichment histogram of DEGs among groups; B: KEGG enrichment bubble plot of DEGs between groups.

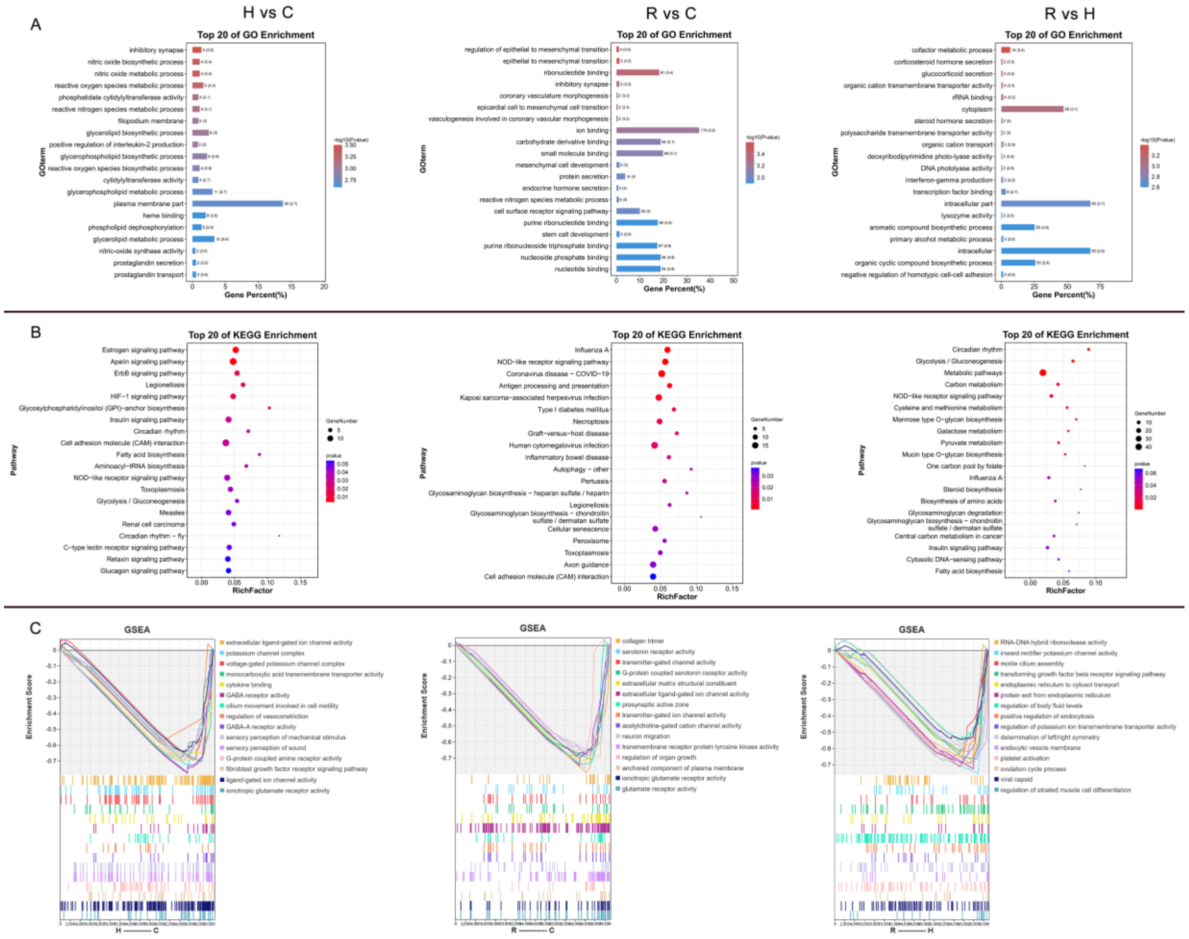

Figure S4. Analysis of DEGs in gill MCs of *T. vachellii*. A: GO enrichment histogram of DEGs among groups; B: KEGG enrichment bubble plot of DEGs between groups; C: GSEA analysis chart of DEGs between groups.

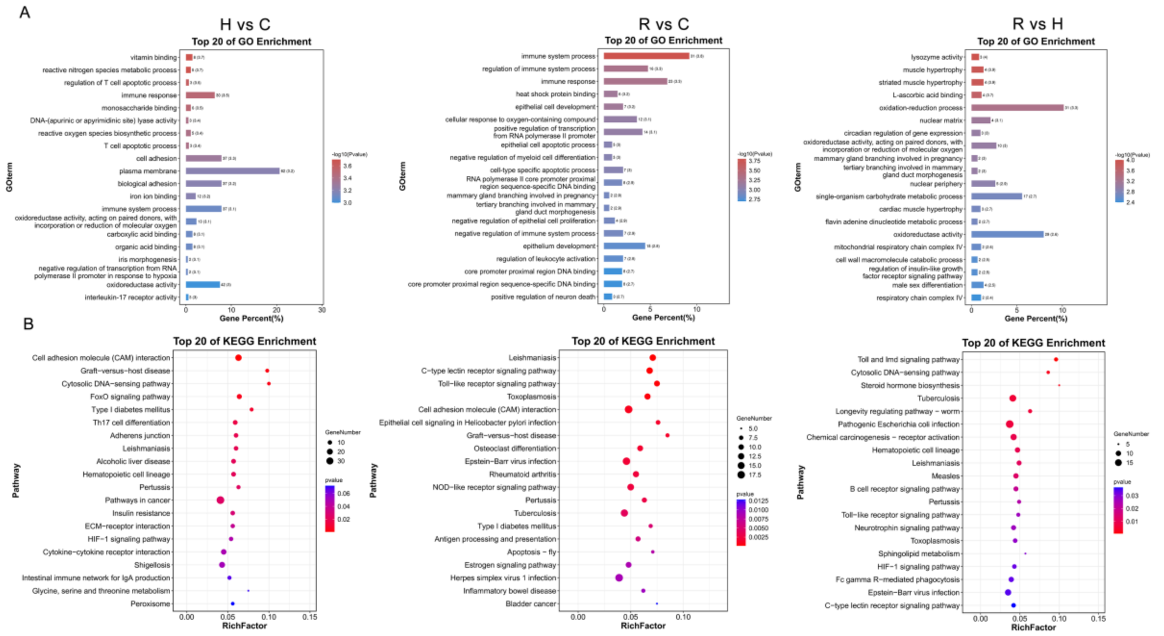

Figure S5. Analysis of DEGs in gill PVCs of *T. vachellii.* A: GO enrichment histogram of DEGs among groups; B: KEGG enrichment bubble plot of DEGs between groups.

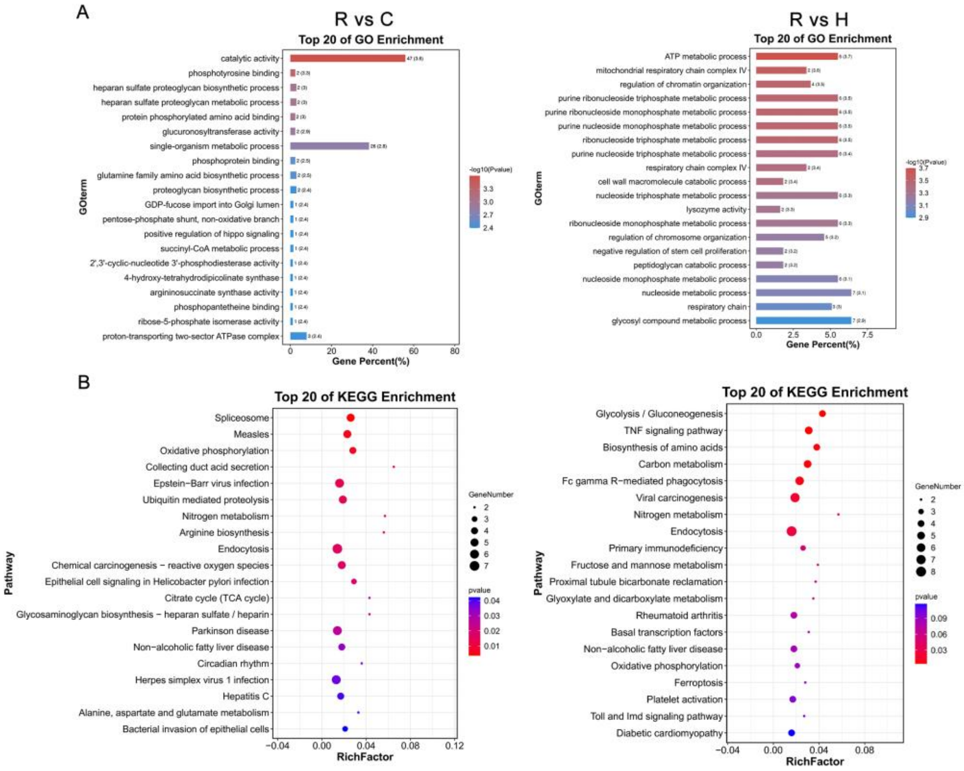

Figure S6. Analysis of DEGs in gill tuft cells of *T. vachelli*. A: GO enrichment histogram of DEGs among groups; B: KEGG enrichment bubble plot of DEGs between groups
